# Extracellular vesicles and their translational therapeutic potential

**DOI:** 10.5599/admet.3328

**Published:** 2026-07-26

**Authors:** Riya Mukherjee, Ramendra Pati Pandey, Chung Ming Chang

**Affiliations:** 1 Graduate Institute of Biomedical Sciences, Chang Gung University, Taoyuan, Taiwan; 2 School of Biosciences and Bioengineering, D Y Patil International University (DYPIU), Akurdi, Pune, Maharashtra, 411044, India; 3 Master & Ph.D. program in Biotechnology Industry, Chang Gung University, No.259, Wenhua 1^st^ Rd., Guishan Dist. Taoyuan City 33302, Taiwan

**Keywords:** Mesenchymal stem cell-derived extracellular vesicles, osteoarthritis therapy, preclinical models, cartilage regeneration, regenerative medicine

## Abstract

**Background and purpose:**

Mesenchymal stem cell-derived extracellular vesicles (MSC-EVs) have emerged as a promising cell-free therapy for osteoarthritis (OA). However, their transition into clinical therapeutics is constrained by unstandardized pharmacokinetics and heterogeneous preclinical reporting.

**Experimental approach:**

A meta-analysis of preclinical in vivo OA models evaluating MSC-EV therapy was conducted. Study quality was rigorously assessed using MISEV 2023 (Minimal Information for Studies of Extracellular Vesicles, 2023) criteria and the SYRCLE (Systematic Review Centre for Laboratory animal Experimentation) risk-of-bias tool. Quantitative synthesis was performed for the Osteoarthritis Research Society International (OARSI) histological score, reporting pooled mean differences (MDs) and 95 % confidence intervals (95% CIs), along with heterogeneity (*I*^2^ statistic) and microRNA (miRNA) cargo analysis.

**Key results:**

MSC-EV administration conferred robust structural protection against cartilage degradation. Quantitative synthesis of human-derived MSC-EVs (26 studies) significantly reduced OARSI scores (MD -3.27, 95% CI -4.66 to -1.88; *p* < 0.0001). Similarly, quantitative synthesis of animal-derived MSC-EVs (8 studies) demonstrated an even more profound effect (MD -5.58, 95% CI -7.13 to -4.03; *p* < 0.0001). Non-parametric Trim-and-Fill analysis estimated zero missing studies in both datasets, confirming that these effect sizes are highly robust to publication bias. Despite these robust efficacy signals, significant heterogeneity (*I*^2^ > 84 %) persisted. Meta-regression revealed that arbitrary dosage metrics, whether reported by particle count or protein concentration, failed to reliably predict treatment efficacy, highlighting severe gaps in dose-exposure standardization.

**Conclusion:**

MSC-EVs demonstrate highly potent, cross-species efficacy in attenuating OA progression. However, clinical translation is critically bottlenecked by a lack of ADMET (absorption, distribution, metabolism, excretion and toxicity) compliance. Future research must prioritize standardized particle-based dosing, in vivo pharmacokinetic tracking, and rigorous cargo-function validation to enable regulatory approval.

## Introduction

Osteoarthritis (OA) is a progressive degenerative joint disorder characterized by the deterioration of articular cartilage, alterations in subchondral bone, joint pain, and reduced mobility [1,2]. Its global impact is profound: as of 2020, approximately 595 million individuals (≈7.6 % of the global population) were affected by OA, with the prevalence increasing by approximately 132 % since 1990 [1]. Conventional treatments, including non-steroidal anti-inflammatory drugs (NSAIDs), intra-articular corticosteroid injections, and ultimately joint replacement, primarily focus on alleviating symptoms rather than altering fundamental disease progression [2]. Consequently, long-term, non-surgical disease modification remains elusive for the vast majority of patients.

Mesenchymal stem cell (MSC) therapy has emerged as a promising regenerative strategy; however, extracellular vesicles (EVs) derived from MSCs are now recognized as a superior, cell-free alternative [3]. MSC-EVs retain the beneficial paracrine effects of their parent cells, delivering chondroprotective microRNAs, lipids, and growth factors while circumventing the risks of cellular senescence, immune rejection, and tumorigenesis [3,4]. While preliminary preclinical models indicate that MSC-EVs effectively mitigate cartilage degradation and suppress inflammatory mediators, their translation into clinical therapeutics is severely bottlenecked by a lack of fundamental pharmacokinetic (PK) and pharmacodynamic (PD) understanding [5]. Currently, EV therapies suffer from heterogeneous isolation methods that lead to variable intra-articular retention times, erratic biodistribution, and unstandardized clearance rates [5,6].

To successfully transition MSC-EVs into disease-modifying biologic agents, their dosing regimens, which are currently arbitrarily defined across the literature by either bulk protein concentration or particle count, must be systematically evaluated [6]. This meta-analysis not only synthesizes the structural efficacy of MSC-EVs in osteoarthritis models but also critically assesses the preclinical landscape through a translational pharmacological lens. By rigorously quantifying effect sizes and evaluating reporting quality against MISEV 2023 standards [7], we aim to highlight critical gaps in dose-exposure relationships, administration frequency, and EV characterization, thereby establishing a benchmark for ADMET (absorption, distribution, metabolism, excretion, and toxicity)-compliant EV therapeutic development.

## Materials and methods

### Protocol and registration

This meta-analysis was conducted in strict adherence to the updated Preferred Reporting Items for Systematic Reviews and Meta-Analyses (PRISMA) 2020 guidelines [8]. Flow diagrams mapping the study selection process were programmatically generated utilizing the PRISMA2020 R package to ensure reproducible reporting [9]. The study protocol was prospectively registered with the International Prospective Register of Systematic Reviews (PROSPERO; registration number CRD420251081490) [10]. The protocol defined the a priori research questions, eligibility criteria, pharmacological data extraction frameworks, and statistical methodologies.

### Literature search and inclusion criteria

A comprehensive systematic search was executed to identify preclinical *in vivo* studies evaluating mesenchymal stem cell-derived extracellular vesicles (MSC-EVs) in animal models of OA. PubMed, Embase, and Web of Science were searched from January 2017 to March 2025. The search strategy combined controlled vocabulary (*e.g.* MeSH terms) and free-text keywords covering “extracellular vesicles”, “exosomes”, “mesenchymal stem cells” and “osteoarthritis”. Eligible studies included controlled *in vivo* OA models utilizing MSC-EV interventions that reported at least one quantitative structural or histological endpoint (*e.g.* Osteoarthritis Research Society International (OARSI) scores). *In vitro* only studies, non-MSC EV sources and articles lacking extractable quantitative data were excluded.

### Data extraction

Data extraction was independently performed using a standardized template, with discrepancies resolved by consensus. To address the recognized heterogeneity in EV isolation and ensure rigorous pharmacological reproducibility, a comprehensive study-by-study methodological matrix was constructed (Supplementary material, Table S1). For each included study, this matrix systematically captures: (1) reagent names and critical solution compositions, (2) instrumentation details (*e.g.* transmission electron microscope (TEM) accelerating voltage, nanoparticle tracking analysis (NTA) models) and (3) specific procedural conditions including centrifugation speeds/relative centrifugal force (RCF), incubation times, temperatures, and injection routes. Where specific parameters were omitted by the original authors, they were explicitly recorded as "not reported" (NR). To align with translational pharmacokinetic (PK) evaluation standards, EV dosing parameters were rigorously extracted. Dose per injection (particles) was used as the primary metric, while protein concentration (μg mL^-1^) was treated as a secondary proxy when particle counts were unavailable. Variables, including injection volume, number of injections, dosing intervals, and follow-up duration, were recorded to evaluate cumulative exposure and intra-articular therapeutic retention.

### Risk of bias and quality assessment

The methodological quality of the included preclinical studies was evaluated using the Systematic Review Center for Laboratory Animal Experimentation (SYRCLE) risk of bias tool [11]. SYRCLE risk of bias domains span selection, performance, detection, attrition and reporting biases (Supplementary Material). Studies were categorized as having a low, high or unclear risk of bias for each domain. Adherence to the Minimal Information for Studies of Extracellular Vesicles (MISEV) 2023 guidelines was also systematically audited for each study [7].

Statistical analysis, meta-regression and publication bias quantitative synthesis was conducted using a random-effects model (REML estimator) to account for the anticipated inter-study heterogeneity arising from varying EV sources, animal models and dosing regimens. OARSI histological score served as the primary endpoint. Because the OARSI score represents a uniform continuous scale across the literature, pooled effect sizes were calculated as mean differences (MDs) with 95 % confidence intervals (CIs). Statistical heterogeneity was evaluated using Cochran’s Q test, the between-study variance (*τ*^2^), and the *I*^2^ statistic, with *I*^2^ > 50 % indicating substantial heterogeneity.

Subgroup analyses were conducted a priori based on (i) experimental animal species, (ii) EV cellular source, and (iii) OA induction model. Mixed-effects meta-regression was utilized to investigate continuous covariates, specifically evaluating whether the administered dosage per injection or the total number of injections moderated the therapeutic effect size.

Potential publication bias was assessed visually using funnel plots and formally quantified using Egger’s regression asymmetry test. To ensure the robustness of our findings against potential small-study effects, a non-parametric Trim-and-Fill analysis was performed to estimate adjusted effect sizes accounting for theoretically missing studies. Furthermore, the fragility of the overall effect was evaluated using Fail-Safe N calculations via the Rosenthal, Orwin, and Rosenberg approaches. A two-sided *p*-value < 0.05 was considered statistically significant for all analyses.

### Compliance with ethical guidelines

This study is a meta-analysis of previously published preclinical studies and did not involve any new animal or human experimentation. Therefore, no additional ethical approval was required.

## Results

### Study characteristics

**Figure 1.**
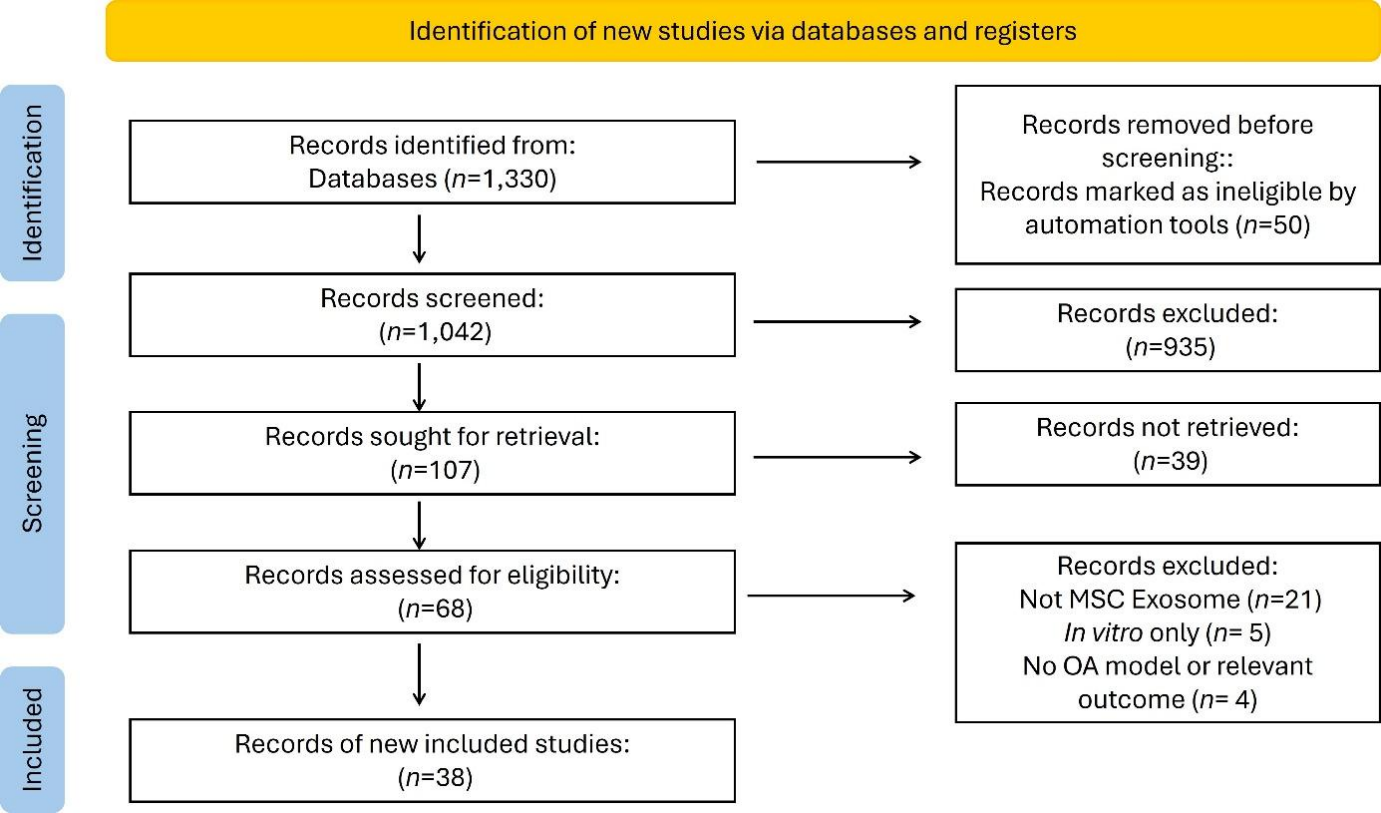
PRISMA flow diagram of the literature search and selection of studies for meta-analysis

Figure 1 provides a summary of the article selection procedure. Initially, 1,330 records were obtained through database searches. After removing duplicates (*n* = 238) and records deemed ineligible by automated screening (*n* = 50), 1,042 unique records were reviewed at the title and abstract level. Following the exclusion of 935 records, 107 articles underwent full-text review. A final cohort of 38 preclinical *in vivo* studies was included in the quantitative synthesis. The key characteristics of the included studies are summarized in Table 1. The 38 studies encompassed both human-derived (28 studies) [12-39] and animal-derived (10 studies) [40-49] MSC-EVs evaluated across diverse preclinical OA models. Of these, 26 human-derived studies and 8 animal-derived studies provided extractable quantitative data for the primary OARSI score meta-analysis. The remaining two animal-derived studies [48,49] met the inclusion criteria and are described in Table 1, but were excluded from the quantitative synthesis because OARSI histological score values (mean ± SD) were not reported in numerical form in the text, tables, or supplementary data of the original publications, precluding mean difference calculation. Rodent models (C57BL/6 mice and Sprague-Dawley rats) predominated, with OA primarily induced via surgical techniques (*e.g.* destabilization of the medial meniscus (DMM), anterior cruciate ligament transection (ACLT)) or chemical induction (*e.g.* monosodium iodoacetate [MIA]) [12-49]. EV cellular origins were highly heterogeneous, including bone marrow, adipose tissue, synovial membrane, umbilical cord, and induced pluripotent stem cells.

**Table 1.** General characteristics included human and animal studies

Study ID	EV source	Tissue origin	EV source species	Experimental subject	Age, week	Sex/*n*	Weight, g	OA model	Isolation method	Characterization method	Ref.
SD1	iMSC-Exo	Induced pluripotent stem cells	Human	C57B/L10 mice	6	Female/35	NR	CIOA	Ultracentrifugation	RPSA, TEM, WB	[12]
	SMMSCs-Exo	Synovial	Human	C57B/L10 mice	6	Female/36	NR	CIOA	Ultracentrifugation	RPSA, TEM, WB	
SD2	SMMSCs-Exo	Synovial	Human	SD rats	~ 12	Male/30	300 to 350	Complete transection of the medial collateral ligament, ACLT and DMM	NR	NR	[13]
SD3	ESCMSCs-Exo	Embryonic	Human	C57BL/6 J	8	NR/32	NR	DMM	Ultracentrifugation	NR	[14]
SD4	IPFPMSC-Exo	infrapatellar fat pad	Human	C57BL/6 mice	9	Male/39	NR	DMM	ExoQuick™ reagent kit and ultrafiltration	TEM, NTA, WB	[15]
SD5	ADSC-EVs	Adipose	Human	SD rats	7	Male/50	200 to 250	MIA	TFF	TEM, NTA, WB, FC, DLS	[16]
		Adipose	Human	C57BL/6 mice	9	Male/NR	20 to 25	DMM	TFF	TEM, NTA, WB, FC, DLS	
SD6	BMSCs-Exo	Bone Marrow	Human	C57BL/6 J mice	6	Male/23	NR	CIOA	Total exosome isolation kit		[17]
SD7	SMMSCs-Exo	Synovial	Human	SD rats	NR	Male/40	200 to 220	DMM	Ultracentrifugation	TEM, NTA, WB	[18]
SD8	SMMSCs-Exo	Synovial	Human	C57 mice	8	Male/20	25 to 30	Complete transection of the medial collateral ligament, ACLT and DMM	Ultracentrifugation	TEM, NTA	[19]
SD9	UCMSCs-Exo	Umbilical cord	Human	SD rats	8	Male/24	NR	ACLT	Commercial kit	TEM,FC	[20]
SD10	ADSCs-Exo	Adipose	Human	BALB/c mice	3	Female/35	NR	ciprofloxacin induced OA	Ultracentrifugation	TEM, DLS, FC	[21]
	BMSCs-Exo	Bone Marrow	Human	BALB/c mice	3	Female/35	NR	ciprofloxacin induced OA	Exocib exosome extraction kit	TEM, DLS, FC	
SD11	BMSCs-Exo	Bone Marrow	Human	SD rats	NR	Male/20	358 ± 5	ACL+MM	Gradient centrifugation	TEM, FC, WB	[22]
SD12	ADSC-EVs	Adipose	Human	ICR (CD-1) mice	8	Female/30	NR	Bilaterally OVX	TFF	TEM, FC	[23]
SD13	SMMSCs-Exo	Synovial	Human	C57BL/6 J mice	10	Male/NR	NR	DMM	Ultracentrifugation	NTA, TEM, WB	[24]
SD14	UCMSCs-Exo	Umbilical cord	Human	SD rats	NR	NR/18	NR	Surgically induced cartilage defect model	NR	TEM, NTA, FC	[25]
SD15	UCMSCs-Exo	Umbilical cord	Human	SD rats	12	Female/40	250 ± 20	ACLT and DMM	Ultracentrifugation		[26]
SD16	ADSCs-Exo	Adipose	Human	SD rats	NR	NR/15	230 to 280	MIA-OA	Ultracentrifugation		[27]
SD17	iPSCs-Exo	PBMS	Human	NZ Rabbits	NR	Female/9	400	ACLT	Sequential ultracentrifugation	TEM, WB, DLS	[28]
SD18	UCMSCs-Exo	Umbilical cord	Human	C57BL/6 mice	8	Male/NR	NR	DMM	Differential centrifugation	TEM, NTA, WB	[29]
SD19	WJMSC-Exo	Umbilical cord	Human	SD rats	8	Male/24	NR	ACLT	Sequential ultracentrifugation	TEM, AFM, SEM, FC	[30]
SD20	ADSC-Exo	Subcutaneous fat (SC)	Human	SD žrats	6	Male/NR	180 to 200	DMM+ACLT	Ultracentrifugation	TEM, NTA	[31]
	ADSC-Exo	Subcutaneous fat (SC)	Human	C57BL/6 J mice	7	Male/NR	18 to 22	DMM	Ultracentrifugation	TEM, NTA	
SD21	ADSC-Exo	Subcutaneous adipose	Human	SD rats	8	Male/30	NR	ACLT	Ultracentrifugation	TEM, NTA	[32]
SD22	UCMSCs-Exo	Umbilical cord	Human	SD rats	NR	Male	350g	ACL rupture-induced OA	Gradient centrifugation	TEM, NTA	[33]
SD23	DPSC-Exo	Dental pulp stem cell	Human	C57BL/6 mice	7	Male/15	20-25g	MIA	Sequential ultracentrifugation	TEM, NTA, WB	[34]
SD24	pExo	Placenta	Human	SD rats	10 to 12	Male/NR	NR	MCLT+MMT	Sequential ultracentrifugation	NTA, WB	[35]
SD25	UCMSCs-Exo	Umbilical cord	Human	SD rats	6 to 8	Male/24	200 ± 20	MIA	NR		[36]
SD26	UCMSCs-Exo	Umbilical cord	Human	SD rats	8	Male/NR	300 to 350	CIOA	TFF	TEM, NTA,FC	[37]
SD27	UCMSCs-Exo	Umbilical cord	Human	C57BL/6 J mice	8 to 12	Male, Female/ NR	NR	CIOA	Ultracentrifugation	NTA, FC	[38]
SD28	UCMSCs-Exo	Umbilical cord	Human	SD rats	12	Male/NR	300 to 350	ACLT + pMMx	Ultracentrifugation	TEM, ZS, WB	[39]
SD1	BMSCs-Exo	Bone marrow	C57BL/6 mice	C57BL/6 mice	3 days	NR/45	NR	CIOA	Ultracentrifugation	NTA, TEM, DLS, FC	[40]
SD2	BMSCs-Exo	Bone marrow	C57BL/6 mice	C57BL/6 mice	NR	NR/60	25 to 30	Surgically induce instability in the lumbar spine	Ultracentrifugation	TEM, BCA, WB	[41]
SD3	BMSCs-Exo	Bone marrow	C57BL/6 mice	SD rats	Adult	Male/36	200 to 250	ACLT+DMM	Ultracentrifugation	TEM, NTA, WB	[42]
SD4	BMSCs-Exo	Bone marrow	SD Rats	SD rats	6	Male/80	190 ± 10	ACL+MCL	Ultracentrifugation	WB	[43]
SD5	BMSCs-Exo	Bone marrow	SD Rats	SD rats	10	Male/24	NR	MIA-OA	Ultracentrifugation	TEM, NTA, WB	[44]
SD6	BMSCs-Exo	Bone marrow	SD Rats	SD rats	NR	Male/24	200 to 220	MIA-OA	ExoQuick-TC™	TEM, NTA	[45]
SD7	BMSCs-Exo	Bone marrow	Rabbit	NZ rabbits	NR	Male/20	2500 ± 500	ACL+MM	NR	TEM, NTA, FC	[46]
SD8	BMSCs-Exo	Bone marrow	C57BL/6 mice	SD rats	NR	NR/40	NR	MCL+ACL+PCL	ExoQuick Extraction Kit	TEM, NTA, WB	[47]
SD9	ADSCs-Exo	Adipose	C57BL/6 mice	C57BL/6 mice	8	Male/102	25 to 30	LFJ OA	NR	TEM, NTA, WB	[48]
SD10	BMSCs-Exo	Bone marrow	SD Rats	SD rats	10	Male/24	220	ACL+MM	Ultracentrifugation	TEM, NTA, WB	[49]

### Quality of included studies

To contextualize the heterogeneity observed across the included studies and to identify systematic gaps in methodological transparency, we performed a structured reporting-quality assessment of all 38 studies against the MISEV 2023 guidelines. Each study was scored across seven domains derived directly from the comprehensive methodological matrix (Supplementary material, Table S2): isolation method, characterization method (MISEV triad of morphology, particle metrics, and protein markers), size distribution, surface marker analysis, functional testing, procedural reporting clarity (centrifugation force, time, temperature, and equipment), and statistical methods. No study achieved complete adherence to MISEV 2023 reporting standards. Substantial adherence was observed in 7 of 38 studies (18.4 %), Partial adherence in 20 of 38 studies (52.6 %), and limited adherence in 11 of 38 studies (28.9 %). Domain-level analysis revealed that the strongest reporting was in surface marker characterization (32/38, 84.2 % adequate) and the MISEV characterization triad (31/38, 81.6 % adequate), reflecting widespread adoption of the basic transmission electron microscopy/nanoparticle tracking analysis/Western blot characterization framework. In contrast, the most pronounced reporting deficits were observed in numeric size distribution reporting, with only 4 of 38 studies (10.5 %) providing explicit modal diameter or size-range values in the methods or results text rather than in figure panels alone, and in procedural reporting clarity, where only 5 of 38 studies (13.2 %) reported all three of relative centrifugal force/time, isolation temperature, and ultracentrifuge or rotor model. Statistical methods were fully specified (test, software, and significance threshold) in only 10 of 38 studies (26.3 %), whereas functional validation, combining both in vivo OA model outcomes and in vitro mechanistic assays, was reported in 14 of 38 studies (36.8 %).

**Figure 2.**
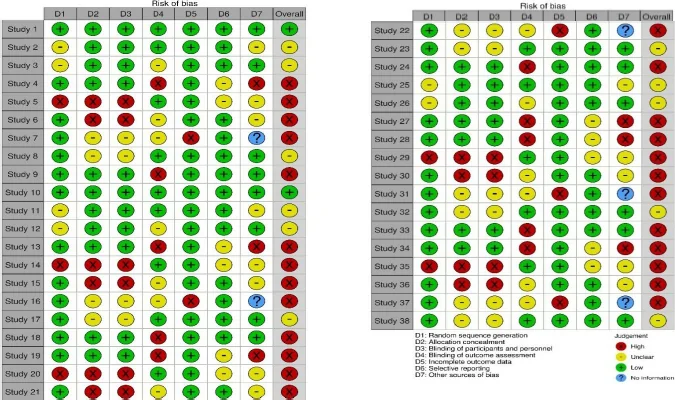
Evaluation of methodological quality of the included studies using the SYRCLE risk of bias tool.

Critical gaps were observed in the reporting of pharmacological dosing parameters. Methodological quality was further evaluated using the SYRCLE risk-of-bias tool (Figure 2). Most studies demonstrated a low risk of bias regarding random sequence generation and selective reporting.

However, blinding of participants/personnel, allocation concealment, and blinded outcome assessments were frequently rated as high or unclear risk, highlighting persistent methodological vulnerabilities in the preclinical EV landscape that may inflate perceived efficacies.

### Effect size (primary outcome)

**Figure 3.**
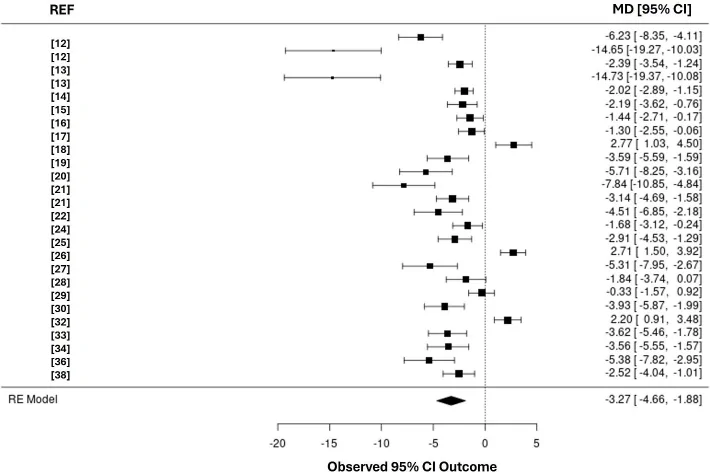
Forest plot of preclinical studies [12-22,24-30,32-34,36,38] evaluating human MSC-derived exosomes in OA models using OARSI histological scoring. The random-effects model (REML) yielded a pooled estimate of -3.27 (95% CI: -4.66 to -1.88; *p* <0.0001), with substantial heterogeneity (*I*^2^ = 94.64%, *τ*^2^ = 11.92, *Q* = 265.59, *df* = 25)

**Figure 4.**
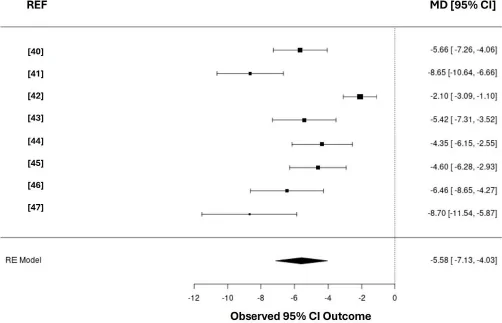
Forest plot of preclinical studies [38-44,46] evaluating animal-derived MSC-derived exosomes in OA models using OARSI histological scoring. The random-effects model (REML) yielded a pooled estimate of -5.58 (95% CI: -7.13 to -4.03; *p* <0.0001), with substantial heterogeneity (*I*^2^ = 84.19%, *τ*^2^ = 4.07, *Q* = 53.37, *df* = 7)

Quantitative synthesis of the OARSI histological score demonstrated robust therapeutic efficacy of MSC-EV administration. For human-derived MSC-EVs, the pooled mean difference (MD) was -3.27 (95% CI: -4.66 to -1.88; *p* <0.0001), indicating significant structural protection of articular cartilage compared with untreated controls. Heterogeneity remained substantial (*I*^2^ = 94.64%, *τ*^2^ = 11.92), reflecting the wide variance in EV sourcing and dosing (Figure 3). Crucially, animal-derived MSC-EVs exerted an even more pronounced protective effect, yielding a pooled MD of -5.58 (95% CI: -7.13 to -4.03; *p* <0.0001). While statistical heterogeneity was also present in the animal-derived subgroup (*I*^2^ = 84.19%, *τ*^2^ = 4.07), the highly significant *p*-value confirms the potent cross-species biological activity of MSC-EVs in mitigating OA progression (Figure 4).

### Dosage characteristics

A focused evaluation of EV dosing revealed profound heterogeneity, underscoring a major barrier to ADMET standardization (Supplementary material, Table S3). Dose per injection (particles) was established as the primary metric, while protein concentration served as a secondary proxy. As summarized in Tables 2 and 3, particle-based dosing was reported in 14 of 38 studies (37 %), spanning 8×10^7^ to 10^10^ particles per injection (median ~3×10^8^; IQR: 10^8^ to 10^9^). Protein-based dosing was reported in 13 of 38 studies (34 %), ranging from 0.25 μg to 500 μg per injection. Critical contextual parameters for pharmacokinetics, such as precise injection volumes, dosing intervals, and intra-articular retention times, were omitted in the majority of reports, thereby preventing precise dose-exposure modelling and highlighting an urgent need for standardization of pharmacological reporting in EV therapeutics.

**Table 2.** Reported dosing regimens of mesenchymal stem cell-derived extracellular vesicles (EVs) in osteoarthritis models

Metric	Median (IQR)	Range	Number of reporting studies
Dose *per* injection (particles), μg	~3×10^8^ (10^8^ to 10^9^)	8×10^7^ to 10^10^	14
Dose *per* injection (protein concentration), μg/mL	40 (10 to 100)	0.25 to 500	13

**Table 3.** An overview of the completeness of dose reporting across included studies

Parameter	Number of reported dose (*n* / %)	Number of not reported dose (*n* / %)
Particles *per* injection	14 (37)	24 (63)
Protein *per* injection	13 (34)	25 (66)

### Subgroup analysis

To delineate drivers of inter-study variance, *a priori* subgroup analyses were conducted for both human-derived and animal-derived MSC-EVs, stratified by EV cellular source, OA induction model and experimental subject.

#### Human-derived MSC-EVs

**Figure 5.**
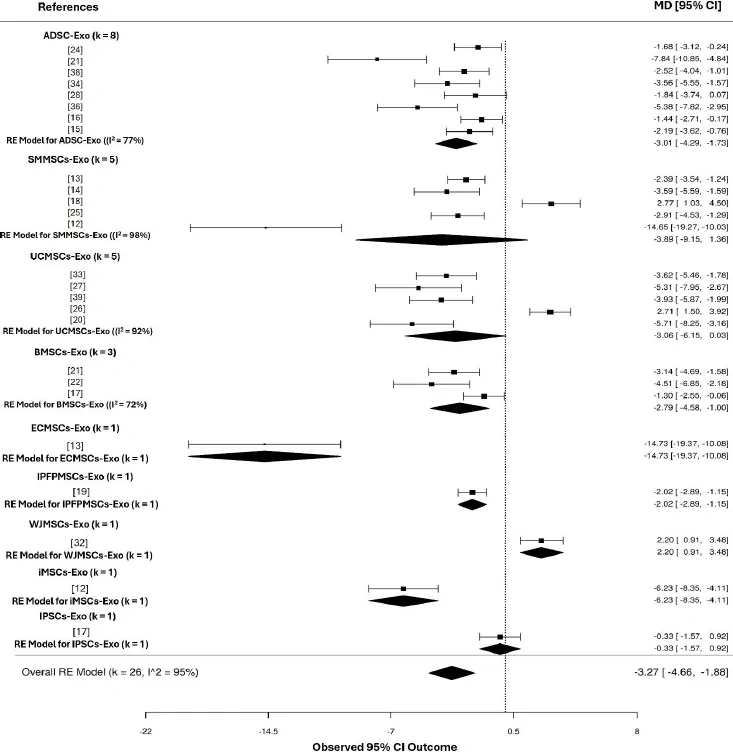
Meta-analysis of EV therapeutic efficacy on OARSI scores subgrouped by tissue origin [12-39]. Forest plot detailing the effect of human-derived extracellular vesicles (EVs) on OARSI scores, stratified by the tissue source of the EVs (*e.g.* ADSC-Exo, SMMSCs-Exo, UCMSCs-Exo, BMSCs-Exo, WJMSC-Exo, iMSC-Exo). The plot displays MD and 95% CI for individual studies and the pooled random-effects (RE) models for each subgroup. The test for subgroup differences indicates no statistically significant variation in therapeutic efficacy based on EV tissue origin (Q_b(8) = 12.79, *p* = 0.119). The overall random-effects model across all studies demonstrates a significant reduction in OARSI scores (MD = -3.27, 95% CI: -4.66 to -1.88)

No significant differences in therapeutic efficacy were observed across tissue origins of the EVs, as indicated by the test for subgroup differences (Q_b(8) = 12.79, *p* = 0.119). Adipose-derived EVs (ADSC-Exo) demonstrated a statistically significant reduction in OARSI scores (MD = -3.01, 95% CI: -4.29 to -1.73). Similarly, bone marrow-derived EVs (BMSCs-Exo) also yielded a significant pooled effect (MD = -2.79, 95% CI: -4.58 to -1.00). Furthermore, induced MSC-derived EVs (iMSC-Exo) showed a strong reduction in scores (MD = -6.23, 95% CI: -8.35 to -4.11). In contrast, Wharton's jelly-derived EVs (WJMSC-Exo) were associated with a positive mean difference (MD = 2.20, 95% CI: 0.91 to 3.48) (Figure 5). The overall random-effects model across all EV types indicated a significant overall reduction in OARSI scores (MD = -3.27, 95% CI: -4.66 to -1.88) (Figure 5).

**Figure 6.**
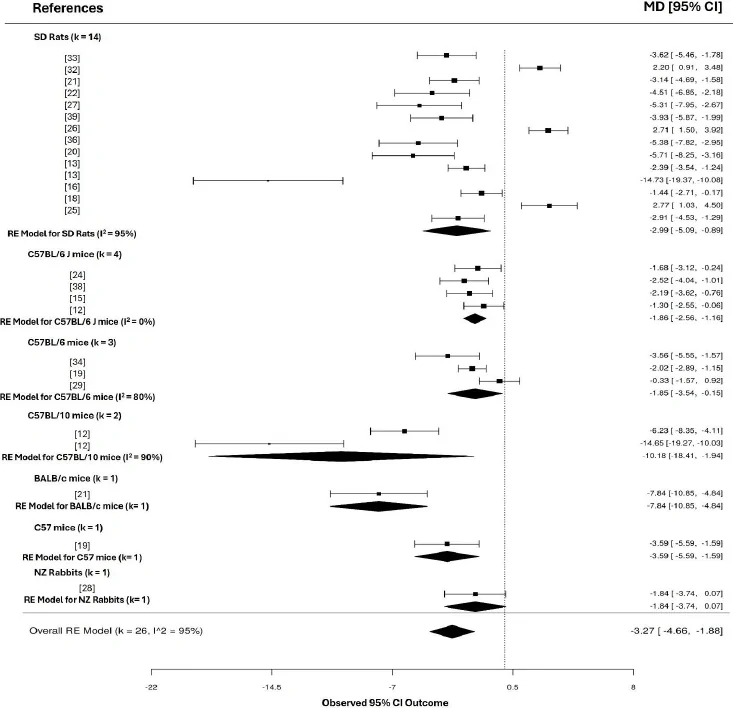
Meta-analysis of EV therapeutic efficacy on OARSI scores subgrouped by experimental subjects [10-37]. Forest plot of a mixed-effects meta-regression assessing the impact of host species and animal models on the therapeutic rescue of EVs, measured via OARSI scores. Subgroups represent the various preclinical xenograft models utilized across studies, including SD rats, multiple strains of mice (*e.g.* C57BL/6 J, C57BL/6, C57B/L10, BALB/c) and NZ rabbits. The analysis indicates that the host species does not significantly moderate the therapeutic efficacy of the EVs (Q_b(6) = 9.08, *p* = 0.169)

A subgroup analysis on experimental subjects (*e.g.* C57 mice, SD rats, NZ Rabbits) indicated that the host species did not significantly moderate the therapeutic efficacy of human-derived EVs (Q_b(6) = 9.08, *p* = 0.169). This suggests that the structural benefits of human MSC-EVs are conserved across various preclinical xenograft models (Figure 6).

**Figure 7.**
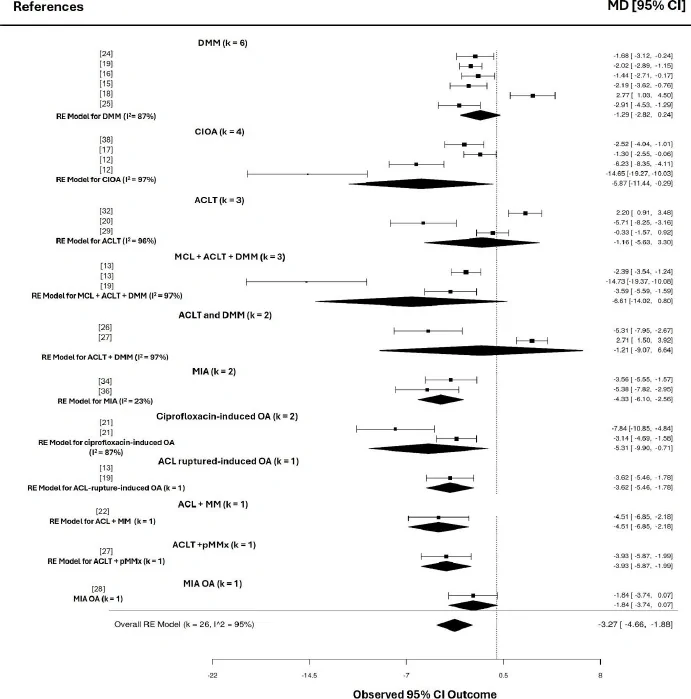
Meta-analysis of EV therapeutic efficacy on OARSI scores subgrouped by OA induction methodology [10-37]. Forest plot evaluating the therapeutic impact of EVs on OARSI scores, stratified by the methodology used to OA in the experimental models. Subgroups include chemically induced models (*e.g.* MIA, CIOA, ciprofloxacin) and surgical models (*e.g.* DMM, ACLT, ACL rupture). The test for subgroup differences reveals no statistically significant variance in therapeutic efficacy across the different induction methodologies (Q_b(10) = 6.62, *p* = 0.761)

Subgrouping by OA induction methodology revealed no statistically significant differences in therapeutic efficacy across the various models, as indicated by the test for subgroup differences (Q_b(10) = 6.62, *p* = 0.761). The MIA model yielded an effect size of MD = -4.33 (95% CI: -6.10 to -2.56). Similarly, Collagenase-Induced OA (CIOA) produced an MD of -5.87 (95% CI: -11.44 to -0.29). Surgical DMM models exhibited high internal variance and a non-significant pooled effect, as the confidence interval crossed zero (MD = -1.29, 95% CI: -2.82 to 0.24) (Figure 7).

#### Animal-derived MSC-EVs

**Figure 8.**
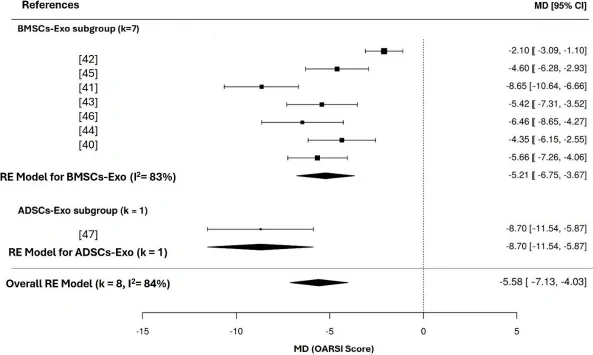
Meta-analysis of animal MSC-EV therapeutic efficacy on OARSI scores subgrouped by EV tissue source [38-44,46]. Forest plot detailing the effect of animal-derived extracellular vesicles (EVs) on OARSI scores, stratified by the tissue origin of the EVs: bone marrow-derived (BMSCs-Exo) and adipose-derived (ADSCs-Exo). The plot displays MD) and 95% CI for individual studies and the pooled random-effects (RE) models. The overall random-effects model across all studies demonstrates a significant reduction in OARSI scores (MD: = -5.58, 95% CI: -7.13, -4.03). The test for subgroup differences indicates no statistically significant variation in therapeutic efficacy between the two tissue sources (Q_b(1) = 1.95, *p* = 0.162)

Consistent with trends in the human subgroup, both adipose- and bone marrow-derived animal MSC-EVs exerted protective effects against cartilage degradation. While bone marrow remains the most frequently investigated source, yielding a highly reproducible significant benefit (MD: -5.21, 95% CI: -6.75 to -3.67), preliminary evidence indicates that adipose-derived EVs may yield an even more potent structural rescue (MD: -8.70, 95% CI: -11.54 to -5.87) (Figure 8).

**Figure 9.**
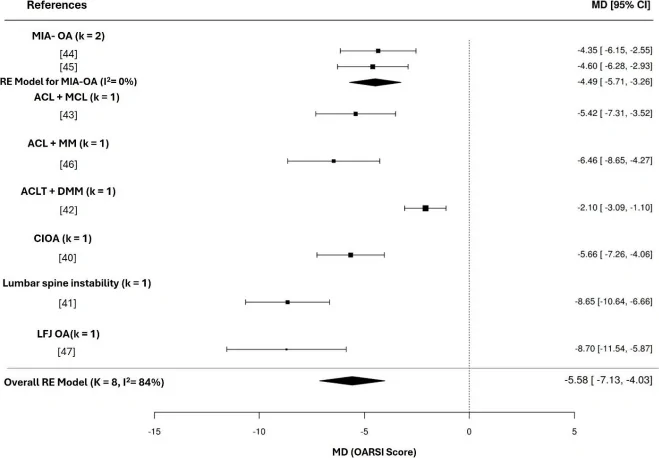
Meta-analysis of animal MSC-EV therapeutic efficacy on OARSI scores subgrouped by OA induction methodology [38-44,46]. Forest plot evaluating the therapeutic impact of animal-derived EVs on OARSI scores across OA induction models. Subgroups include chemically induced models (*e.g.* MIA-OA, CIOA) and multiple surgical instability models (*e.g.* ACL+MCL, ACL+MM, ACLT+DMM, Lumbar spine instability, LFJ OA). While the test for subgroup differences yields a statistically significant p-value (Q_b(6) = 53.33, *p* = 0.000), a notation clarifies that because 6 out of the 7 subgroups consist of only a single study (*k* = 1), the between-model comparison is descriptive rather than inferential. The overall model demonstrates a pooled MD of -5.58 (95% CI: -7.13, -4.03). Note: With *k* = 1 in 6 of 7 subgroups, between-model comparison is descriptive rather than inferential

Therapeutic efficacy varies across induction methods. The chemically induced MIA-OA model yielded a highly significant effect size (MD: -4.49, 95% CI: -5.71 to -3.26), while specific surgical instability models, such as ACL+MM and LFJ OA, demonstrated even more profound improvements (MD: -6.46 and -8.70, respectively) (Figure 9).

**Figure 10.**
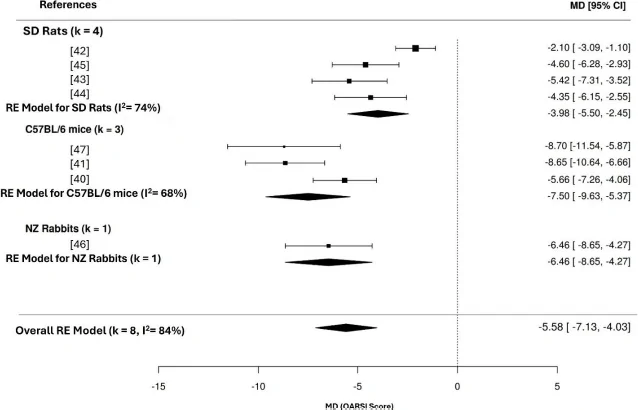
Meta-analysis of animal MSC-EV therapeutic efficacy on OARSI scores subgrouped by experimental host species [38-44,46]. Forest plot assessing the impact of different preclinical animal hosts on the therapeutic rescue of EVs, measured via OARSI scores. Subgroups represent the host species utilized across the evaluated studies, which include SD rats, C57BL/6 mice and NZ rabbits. The test for subgroup differences indicates that the choice of experimental host significantly moderates the therapeutic efficacy of the EVs (Q_b(2) = 7.55, *p* = 0.023). The overall random-effects model reflects a significant therapeutic benefit across all subjects (MD = -5.58, 95% CI: -7.13, -4.03).

Animal-derived EVs successfully attenuated cartilage degeneration across diverse hosts, including C57BL/6 mice (MD: -7.50, 95% CI: -9.63 to -5.37), SD rats (MD: -3.98, 95% CI: -5.50 to -2.45), and NZ rabbits (MD: -6.46, 95% CI: -8.65 to -4.27). Efficacy was preserved across both small-rodent and larger-animal hosts, reinforcing the robust translational and cross-species potential of MSC-EV therapies (Figure 10).

### Evaluation of publication bias and sensitivity analysis

**Figure 11.**
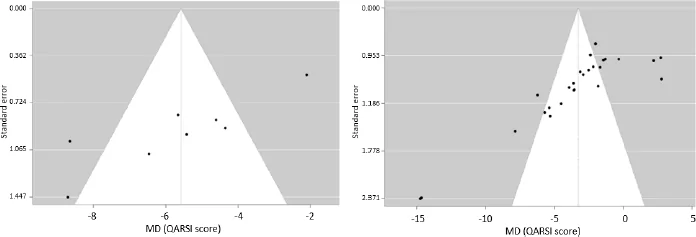
Funnel plots assessing publication bias and small-study effects. (A) Funnel plot of 26 preclinical studies evaluating human-derived MSC-EVs on OARSI scores. Visual asymmetry indicates the presence of small-study effects, formally corroborated by Egger's regression test (*z* = -7.50, *p* <0.0001). (B) Funnel plot of 8 studies evaluating animal-derived MSC-EVs. Similar asymmetry is observed, confirming significant small-study effects (*z* = -4.56, *p* <0.0001)

Potential publication bias and small-study effects were initially evaluated using Egger’s regression test for funnel plot asymmetry. Highly significant asymmetry was detected across both datasets, with human-derived MSC-EVs (*z* = -7.50, *p* <0.0001) and animal-derived MSC-EVs (*z* = -4.56, *p* <0.0001) demonstrating a departure from symmetry (Figure 11).

To rigorously assess whether this potential publication bias invalidated our primary findings, we performed a nonparametric Duval and Tweedie Trim-and-Fill analysis. For both the animal-derived dataset (*k* = 8) and the human-derived dataset (*k* = 26), the model estimated 0 missing studies on the right side. Consequently, the imputed models did not alter the original effect sizes, confirming the robustness of the significant therapeutic benefits observed for both animal-derived (MD = -5.58, 95% CI: -7.13 to -4.03) and human-derived EVs (MD = -3.27, 95% CI: -4.66 to -1.88).

To further test the stability of these findings against unpublished null results, Fail-safe *N* calculations were conducted. The Rosenthal approach indicated that 2,058 and 813 missing studies with an effect size of zero would be required to nullify the statistical significance of the human and animal datasets, respectively. Similarly, the Rosenberg Fail-safe *N* corroborated this high tolerance, requiring 906 and 485 missing studies to bring the significance level above. Finally, the Orwin approach demonstrated that 26 and 8 missing studies would be needed to reduce the respective average effect sizes exactly in half. Together, these sensitivity analyses indicate that although funnel plot asymmetry is present, the observed protective effects of MSC-EVs on cartilage integrity remain statistically robust and are highly unlikely to be overturned by unpublished negative data.

## Discussion

This meta-analysis of 38 preclinical studies provides robust quantitative evidence that mesenchymal stem cell-derived extracellular vesicles (MSC-EVs) substantially attenuate osteoarthritic cartilage degeneration. In human-derived EVs, the pooled MD for OARSI histological scores was -3.27 (95 % CI: -4.66 to -1.88). Animal-derived EVs demonstrated an even more profound structural rescue, yielding an MD of -5.58 (95 % CI: -7.13 to -4.03). Importantly, the magnitude of these effects is clinically meaningful when interpreted against the global burden of disease: 595 million individuals (≈ 7.6 % of the world’s population) lived with osteoarthritis (OA) in 2020, with case numbers increasing by approximately 132 % since 1990 [1]. Rigorous publication-bias assessments support the validity of our pooled estimates: Trim-and-Fill analyses estimated zero missing studies across both datasets, and Rosenthal Fail-Safe *N* values of 2058 (human) and 813 (animal) establish that these therapeutic effect sizes are highly resistant to the file-drawer problem.

Despite these robust efficacy signals, the pervasive heterogeneity observed across studies (*I*^2^ > 84 %) presents a critical translational barrier that must be examined through a pharmacokinetic (PK) and ADMET lens. EV therapies in preclinical OA models currently suffer from unstandardized dosing regimens arbitrarily defined by either protein concentration (ranging from 0.25 to 500 μg mL^-1^) or particle count (8×10^7^ to 10^10^ particles). As our meta-regression indicated, arbitrary dosage metrics alone fail to significantly predict treatment efficacy. This is consistent with established intra-articular (IA) PK behaviour: small-molecule drugs and protein biologics injected into the synovial cavity are typically cleared with half-lives of only a few hours, owing to rapid synovial-fluid turnover and extensive lymphatic and venous drainage [37]. Free MSC-EVs delivered IA face the same clearance pressures, compounded by macrophage uptake within the synovial lining. Reporting a dose without defining the joint retention time, cumulative exposure (AUC), or biodistribution is therefore pharmacologically incomplete and obscures meaningful comparisons between studies.

Subgroup analyses revealed distinct pharmacodynamic profiles based on EV cellular origin. While bone marrow-derived EVs (BMSC-EVs) provided highly reproducible benefits across species, adipose-derived EVs (ADSC-EVs) exhibited particularly potent structural rescue, yielding MDs of -3.01 and -8.70 in human and animal subgroups, respectively. This differential efficacy is plausibly driven by variation in EV miRNA cargo (Table S5). Among MSC-derived EV miRNAs, miR-140-5p has been most directly linked to chondroprotection: synovial-MSC exosomes engineered to overexpress miR-140-5p enhanced articular chondrocyte proliferation and migration and prevented OA progression in a rat model, indicating cargo-specific potency rather than a generic vesicle effect [11]. The frequently reported chondroprotective miRNAs (*e.g.* miR-100-5p, miR-140-5p, miR-26a-5p) converge on extracellular-matrix metabolism and mTOR/autophagy pathways. However, in the absence of standardized potency assays mapping specific cargo concentrations to target engagement, the precise dose-response relationship for any individual miRNA cargo remains elusive across the included studies.

Methodological rigor across the included studies remains a significant vulnerability and likely contributes substantially to the heterogeneity observed in our pooled estimates. Although most studies adhered to the basic characterization triad endorsed by MISEV 2023, morphology by transmission electron microscopy, particle quantification by nanoparticle tracking analysis, and a small panel of tetraspanin markers, MISEV 2023 emphasizes that adequate EV characterization now requires multi-modal verification across morphology, particle metrics, and protein/lipid composition, together with full reporting of separation conditions [7]. Our formal MISEV 2023 adherence assessment of all 38 included studies (Table S2) revealed that no study achieved Complete adherence, and only 18 % reached Substantial adherence; the remaining 82 % were classified as Partially or Limitedly adherent. The two most pronounced reporting deficits, numeric size distribution data (adequate in only 11 % of studies) and procedural reporting clarity covering centrifugation force, temperature, and equipment (adequate in only 13 %), directly compromise reproducibility and prevent meaningful between-study comparison of EV preparations. The omission of exact ultracentrifugation speeds, temperatures, and critical solution compositions in many original reports threatens reproducibility, and the lumping of distinct vesicular populations (small EVs versus larger microvesicles) due to unstandardized isolation inevitably confounds efficacy readouts and downstream regulatory oversight. These findings provide quantitative support for the field-wide call to make MISEV 2023 compliance a standard prerequisite for publication of EV therapeutic studies.

To transition MSC-EVs from promising experimental biologics to regulated advanced therapy medicinal products (ATMPs), the field must pivot toward rigorous ADMET compliance and harmonized manufacturing. EV therapeutics already fall under the regulatory umbrella of established biologics frameworks: in the United States, EV products require Investigational New Drug filings to FDA’s CBER or CDER, and in the European Union the EMA classifies EV-based medicines as ATMPs under Regulation (EC) No 1394/2007, with sponsors filing Clinical Trial Applications *via* the centralised Clinical Trials Information System and obtaining Committee for Advanced Therapies classification [49-51]. Meeting these standards requires moving beyond simple efficacy readouts toward in vivo imaging that quantifies intra-articular EV half-life and biodistribution, evaluation of hydrogel or scaffolding delivery systems to prolong the therapeutic window, and the establishment of standardized particle-to-biomolecule ratios for dosing. Only through methodological harmonization and integrated PK/PD modelling can MSC-EVs achieve the clinical reliability required for true disease modification in osteoarthritis.

## Conclusion

This meta-analysis establishes the definitive structural efficacy of mesenchymal stem cell-derived extracellular vesicles (MSC-EVs) in preclinical osteoarthritis models, demonstrating profound and highly robust cartilage protection across both human- and animal-derived sources. Crucially, rigorous publication bias analyses confirm these therapeutic signals are not artifacts of selective reporting. However, the pervasive methodological heterogeneity observed, specifically the reliance on arbitrary and incomplete dosing metrics, represents a critical translational barrier. The path forward for MSC-EV therapeutics does not lie in accumulating further basic efficacy data, but rather in adopting stringent ADMET and pharmacokinetic frameworks. By standardizing isolation procedures and reporting, harmonizing particle-to-biomolecule dosing ratios, and integrating precise in vivo clearance and biodistribution tracking, the field can successfully transition MSC-EVs from promising experimental biologics to safe, reproducible, and regulatory-approved advanced medicinal products (ATMPs).

## Supplementary material

Additional data are available at https://pub.iapchem.org/ojs/index.php/admet/article/view/3328, or from the corresponding author on request.


