# Supplementary material for: Extracellular vesicles and their translational therapeutic potential
Source: ADMET DMPK. 2026 Jul 26;14:3328. doi: 10.5599/admet.3328 (PMC13499667; doi:10.5599/admet.3328)
Supplement: Supplementary file 1 [file ADMET-14-3328-S1.pdf]

Open Access : ISSN : 1848-7718
http://www.pub.iapchem.org/ojs/index.php/admet

Supplementary material to

Extracellular vesicles and their translational therapeutic potential

Riya Mukherjee[1], Ramendra Pati Pandey[2] and Chung Ming Chang[3]

[1]*Graduate Institute of Biomedical Sciences, Chang Gung University, Taoyuan, Taiwan*
[2]*School of Biosciences and Bioengineering, D Y Patil International University, Akurdi, Pune, India*
[3]*Master & Ph.D. program in Biotechnology Industry, Chang Gung University, No.259, Wenhua 1st Rd., Guishan Dist. Taoyuan City 33302, Taiwan*

ADMET & DMPK **14** (2026) 3328; https://doi.org/10.5599/admet.3328

Materials and methods

Literature search and inclusion criteria

A comprehensive and systematic search strategy was employed to identify relevant preclinical studies assessing the role of extracellular vesicles (EVs) in animal models of osteoarthritis (OA). Searches were conducted in PubMed, Embase, and Web of Science databases for studies published between January 2017 and March 2025, using a combination of controlled vocabulary terms and free-text keywords related to “extracellular vesicles”, “exosomes”, “mesenchymal stem cells”, and “osteoarthritis.” Boolean operators were applied to combine search terms. To ensure thorough coverage, the reference lists of relevant primary studies and review articles were also screened manually to identify additional eligible studies. Studies were included if they involved *in vivo* preclinical models of OA, used MSC-derived EVs as an intervention, incorporated a suitable control group, and reported at least one extractable quantitative outcome related to OA progression, such as histological scoring, structural integrity, or molecular markers of cartilage degradation. Exclusion criteria comprised studies restricted to *in vitro* experiments, clinical studies, review articles, conference abstracts, and those lacking quantitative extractable data. Title and abstract screening, followed by full-text review, was conducted independently by two investigators, and disagreements were resolved through discussion until consensus was reached.

Data extraction

Data extraction was carried out using a standardized Microsoft Excel template to ensure consistency across studies. Two investigators independently extracted data, and all entries were cross-verified for accuracy. Extracted information included study characteristics (first author, year of publication, animal species, OA induction method), and reported outcome measures (histological scores such as OARSI, and functional readouts). Crucially, to adhere to MISEV 2023 guidelines and

ensure pharmacological reproducibility, a specialized methodological matrix was extracted for EV parameters. This matrix systematically captured: (1) Reagent names, concentrations, and manufacturer details; (2) Critical solution compositions; (3) Instrumentation models and parameters (*e.g.* TEM accelerating voltages, NTA camera settings) and (4) exact procedural conditions including centrifugation speeds (relative centrifugal force [RCF]), durations, and temperatures. Where the original authors failed to provide these critical parameters, they were explicitly coded as not reported (NR) to highlight methodological gaps in the current literature.

Evaluating the risk of bias

The methodological quality and risk of bias of the included studies were assessed independently by two investigators using SYRCLE's risk of bias tool, which comprises 10 predefined domains:

1. Sequence generation
2. Baseline characteristics
3. Allocation concealment
4. Random housing
5. Blinding against performance bias
6. Random outcome assessment
7. Blinding against detection bias
8. Incomplete outcome data
9. Selective outcome reporting
10. Other potential sources of bias (*e.g.* contamination or undue influence of funders)

For each domain, risk of bias was judged as low, high, or unclear based on the following signaling questions:

Q1: Was the allocation sequence adequately generated and applied?

Q2: Were the groups comparable at baseline, or were confounders appropriately adjusted for in the analysis?

Q3: Was allocation adequately concealed?

Q4: Were animals randomly housed during the experiment?

Q5: Were caregivers and/or investigators blinded to group allocation during the experiment?

Q6: Were animals selected at random for outcome assessment?

Q7: Was the outcome assessor blinded?

Q8: Were incomplete outcome data adequately addressed?

Q9: Is the study report free from selective outcome reporting?

Q10: Was the study free of other potential risks of bias?

CC BY

A response of “yes” indicated low risk of bias, “no” indicated high risk of bias, and “unclear” was used when insufficient information was provided. As recommended by SYRCLE’s guidelines, we emphasized assessing the risk of bias rather than generating an overall quality score for each study.

Statistical methods

Visual asymmetry in funnel plots was formally quantified using Egger’s regression. Because significant asymmetry was detected, we employed the non-parametric Duval and Tweedie Trim-and-Fill method. This iterative procedure estimates the number of theoretically 'missing' studies required to create funnel plot symmetry, imputes their effect sizes, and recalculates an adjusted pooled effect estimate to test whether the original finding was merely an artifact of publication bias. Furthermore, the fragility of the overall effect size was audited using Fail-Safe N calculations via the Rosenthal, Orwin, and Rosenberg approaches, determining the absolute number of null-effect studies that would be required to reduce the observed significant effect size to a statistically non-significant level ($p > 0.05$).

Table S1. Comprehensive study-by-study methodological matrix of all included MSC-EV studies (*n* = 38) for the meta-analysis. Items not reported in the original publication are indicated as NR (not reported). Each NR entry was verified by independent inspection of the full-text publication; the parameter was genuinely absent from the methods, results and supplementary sections of the original study.

Human-derived MSC-EV studies (*n* = 28)

Cell passage at EV harvest	EV priming / genetic modification	EV production medium (composition)	Condition-ing time, h	EV Isolation method	Isolation protocol - RCF / time / steps	Isolation equipment (rotor, ultra-centrifuge model)	Isolation tempe-rature	Commercial kit (catalog/manufac turer if used)	Characterisatio n techniques (overview)	TEM Instrument (model + accelerating voltage)	NTA / particle sizer (model + manufacturer)	DLS / Zetasizer instrument	Western Blot markers + antibodies (clone, manufacturer, catalog)	Ethics approval / regulatory ID	Statistical methods	Ref.
MSCs at ~ 80 % confluency	None	MesenGro hMSC medium (StemRD, San Francisco, CA, USA) for production phase; baseline DMEM + 10 % FBS + 2 mM L-glutamine + 1 % pen/strep + 0.1 mM NEAA (Gibco)	48	Ultrafiltration (Note: Table 1 lists 'Ultracentri-fugation' - paper text describes ultrafiltration approach)	300g 10 min / 1,500g 10 min at 4 °C / 0.22 µm filter (Steritop, Millipore) / Ultra-clear tube (Millipore) / 4,000g until upper compartment / 200 µL / resus-pended in PBS / re-ultrafiltered at 4,000g to 200 µL ×2 cycles. Stored at 80 °C.	Ultra-clear tubes (Millipore); centrifuge model NR	4 °C	Not used	TRPS (qNano), TEM, Western blot, BCA	FEI Tecnai G2 spirit TEM (FEI, Eindhoven, Netherlands); accelerating voltage NR	Tunable Resistive Pulse Sensing (TRPS) by qNano (Izon Science, Cambridge, MA); Nanopore NP150 (A37355) at 47.0 mm stretch, 0.6 V; Izon Control Suite v2.2	Not performed	Anti-CD9 (1:1000; Abcam, Cambridge, UK); anti-CD63 (1:1000; Abcam); anti-TSG101 (1:1000; Santa Cruz)	Ethics Committee of Shanghai Jiao Tong University Affiliated Sixth People's Hospital (Approval Number: YS-2016-063); animal SYXK 2011-...	Mann-Whitney U test, unpaired Student's t test; $p < 0.05$; software not specified	[1]
NR	Lentiviral overexpression of miR-140-5p (vs. non-overexpressing SMSCs control)	DMEM/F12 (Hyclone) + 10 % FBS + penicillin/ streptomycin + 250 ng mL^{-1} amphotericin B (Gibco)-chondrocyte medium described; specific exosome production medium NR in main text	NR (deferred to supple-mentary methods)	NR (described as 'isolated from conditioned medium of SMSCs'; specific protocol deferred to supplementary methods)	NR in main text — paper states details described in supplementary methods'	NR	NR	Total Exosome RNA & Protein Isolation Kit (Invitrogen, Carlsbad, CA, USA) — used for downstream RNA/protein extraction, not vesicle isolation	DLS, TEM (per main text); main text does not detail TEM/DLS instruments	NR (TEM used; model not specified in main text)	Not performed (DLS used instead)	DLS used; instrument NR	Anti-CD63, anti-CD9, anti-CD81, anti-Alix (System Biosciences); BCA reagents from Cell Signaling Technology / Abcam	Ethical Committee of Shanghai Jiao Tong University; ARRIVE guidelines compliant	Student's t-test; SPSS 16.0 (IBM); $p < 0.05$	[2]

Cell passage at EV harvest	EV priming / genetic modification	EV production medium (composition)	Condition-ing time, h	EV Isolation method	Isolation protocol - RCF / time / steps	Isolation equipment (rotor, ultra-centrifuge model)	Isolation tempe-rature	Commercial kit (catalog/manufac turer if used)	Characterisatio n techniques (overview)	TEM Instrument (model + accelerating voltage)	NTA / particle sizer (model + manufacturer)	DLS / Zetasizer instrument	Western Blot markers + antibodies (clone, manufacturer, catalog)	Ethics approval / regulatory ID	Statistical methods	Ref.
NR	None	NR fully (conditioned medium of ESC-MSCs; details in main text limited)	NR	Sequential / multistep ultracentrifu-gation	Multistep ultracentrifugatio n as previously described (per text); specific RCF/time values deferred to referenced protocol	NR (specific UC model NR in extracted text)	NR	Not used	TEM (formvar/carbon-coated grids); WB	NR (TEM; method via formvar/carbo n-coated grids)	NR in extracted text	NR	Rabbit anti-CD9 (1:2000; Abcam Cat. ab92726); mouse anti-CD63 (1:200; Santa Cruz); markers per Table 1 also: 'NR'	Ethics Committee of Zhejiang University	One-way ANOVA with Tukey-Kramer *post-hoc*; Mann-Whitney; SPSS; GraphPad Prism for plots	[3]
NR	None	NR in extracted summary	NR	ExoQuick™ reagent kit + ultrafiltration (*per* Table 1)	ExoQuick™ reagent kit (System Biosciences) followed by ultrafiltration step; specific RCF/time values NR in extracted summary	NR	NR	ExoQuick™ kit (System Biosciences, USA)	TEM, NTA, WB (per Table 1)	NR in extracted text	NR in extracted text	Not performed (NTA used)	WB confirmed (markers NR in extracted summary; conventional CD9/CD63/CD81/T SG101 expected per field)	NR in extracted summary (institutional approval implied)	NR in extracted summary	[4]
NR in extracted summary	None	NR in extracted summary	NR	Tangential flow filtration (TFF)	TFF system applied to conditioned medium; specific RCF/time values NR in extracted summary	TFF system (NR specific make/model in extracted text; Lee 2021 from same group used Pall Minimate)	NR	Not used (TFF system rather than precipitation kit)	TEM, NTA, WB, Flow cytometry (FC), DLS (per Table 1)	NR in extracted text	NR specific model in extracted text	DLS performed; instrument model NR in extracted text	Markers NR specifically in extracted summary; WB performed	NR in extracted summary	NR in extracted summary	[5]
NR	None (comparison of pBMSCs vs BMSCs as cellular source)	NR specific composition in extracted summary	NR	Total Exosome Isolation Kit (Invitrogen)	Per manufacturer's protocol: Total Exosome Isolation Kit (Invitrogen) applied to conditioned medium after 300*g* 10 min then further centrifugation	NR	NR	Total Exosome Isolation Kit (Invitrogen)	TEM (FEI Tecnai G2 spirit); WB (*per* Table 1: NR but text confirms WB for CD9/CD63/ /TSG101)	FEI Tecnai G2 spirit transmission electron microscope	NR in extracted text	NR	Anti-CD9 (1:1000; Abcam, Cambridge, UK); anti-CD63 (1:1000; Abcam); anti-TSG101 (1:1000; Santa Cruz)	Ethics Committee of Soochow University (Approval No. SUDA20200-707H01)	SPSS v18 (SPSS, Chicago, IL, USA); Student's *t*-test (2 groups); ANOVA (>2 groups)	[6]
NR	NR	NR	NR	Ultracentrifuga-tion (*per* Table 1)	NR	NR	NR	Not used (*per* Table 1: ultracentrifugation)	TEM, NTA, WB (*per* Table 1)	NR	NR	NR	NR	NR	NR	[7]
NR	miR-31 mimic or inhibitor transfection of SMSCs / chondrocytes	NR specific composition in extracted summary	NR	Ultracentrifugatio n (single-step described)	Ultracentrifugatio n at 100,000*g* for 20 min as final step; pre-clearing steps NR specifically in extracted text	NR	NR (4 °C implied)	Not used	TEM, NTA (*per* Table 1); WB	NR in extracted text	NR in extracted text	Not performed	Anti-CD81, anti-CD9, anti-CD63 (markers expressed); anti-calnexin (negative control marker for non-EV); MSC markers CD44, CD90, CD105 positive; CD14, CD34, CD45 negative	Ethics Committee of the Fourth Hospital of Harbin Medical University	Not specified in extracted text	[8]

(cc) BY

Cell passage at EV harvest	EV priming / genetic modification	EV production medium (composition)	Conditioning time, h	EV Isolation method	Isolation protocol - RCF / time / steps	Isolation equipment (rotor, ultracentrifuge model)	Isolation temperature	Commercial kit (catalog/manufacturer if used)	Characterisation techniques (overview)	TEM Instrument (model + accelerating voltage)	NTA / particle sizer (model + manufacturer)	DLS / Zetasizer instrument	Western Blot markers + antibodies (clone, manufacturer, catalog)	Ethics approval / regulatory ID	Statistical methods	Ref.
NR	None (comparing hUC-MSCs *vs.* hUC-MSC-sEVs)	Exosome-free FBS (MSCYF01-500, YINFENG BIOLOGICAL, China)	NR	Differential ultracentrifugation + ultrafiltration	0.22 µm filter to remove cell debris / supernatant transferred to 100 kDa ultrafiltration tube (UFC9100, Millipore) / conventional differential ultracentrifugetion / resuspended in PBS	100 kDa ultrafiltration tube UFC9100 (Millipore)	NR	Not used (UF + UC approach)	TEM, FC (per Table 1)	NR in extracted text	NR (Table 1 lists FC instead of NTA)	Not performed	FITC anti-human CD63 (BD #556019); FITC anti-human CD9 (BD #555371); FITC anti-human CD81; mesenchymal markers CD105, CD73, CD90 (positive); CD34, CD45, HLA-DR (negative)	Institutional Animal Ethics Committee of Fujian Medical University, Fuzhou, Fujian, China (FJMU IACUC 2020–...)	One-way ANOVA + nonparametric tests in IBM SPSS	[9]
NR	None	NR specific composition in extracted summary	NR	ADSC arm: ultracentrifugation; BMSC arm: Exocib Exosome Extraction Kit (*per* Table 1)	ADSC: ultracentrifugation (specifics NR in extracted summary); BMSC: per Exocib kit protocol	Beckman FC500 flow cytometer used for cell phenotyping (different from EV isolation)	NR	Exocib Exosome Extraction Kit (BMSC arm only; ADSC arm uses UC)	TEM, DLS, FC (*per* Table 1)	NR in extracted text	Not performed (DLS instead)	DLS performed; instrument NR in extracted text	MSC markers CD90, CD73, CD105 (positive >80%); EV markers CD63 and CD81 analyzed; FC500 flow cytometer (Beckman Coulter)	Ethics committee approval at Academic Center for Education, Culture, and Research (ACECR), Qom Branch	ANOVA with multiple comparisons test	[10]
Passage 3 (cells used at P3 for characterization)	None (comparison with cardiac homing peptide [CHP]–targeted exosomes)	Low-glucose DMEM (Gibco, MD) + penicillin/streptomycin (Gibco)	NR	Gradient / sequential ultracentrifugation	1,000*g*, 10 min / 2,000*g*, 10 min / 10,000*g*, 30 min; supernatant retained and ultracentrifuged at 100,000*g* for 70 min; 50 mL conditioned medium starting volume	NR specific UC model	NR (4 °C implied)	Not used	TEM, FC, WB (*per* Table 1)	NR (TEM via glutaraldehyde + 2 % PFA in cacodylate buffer fixation)	Not performed	Not performed	Anti-CD9, anti-CD63 (positive), anti-calnexin (negative)	Affiliated Hospital of Nantong	Student's t-test (2 groups); one-way ANOVA (>2 groups) $p < 0.05$	[11]
NR	None	NR specific composition in extracted summary	NR	Tangential flow filtration (TFF)	Multi-filtration system based on TFF (Minimate TFF System; Pall Corporation, Port Washington, NY, USA), based on Woo et al., 2020 protocol; CM 500 mL processed through TFF	Minimate TFF System (Pall Corporation, Port Washington, NY, USA)	NR	Not used (TFF instead)	TEM, DLS, zeta potential, FC (microbead-based), micro-BCA, NTA (per text)	NR (TEM used)	LM-... (NanoSight; specific model truncated in extracted text)	DLS performed; instrument NR specifically	Microbead-based FC: anti-CD9, anti-CD63, anti-CD81 (Immunostep); anti-GM130 (Novus Biologicals); anti-calnexin (Novus Biologicals); EVs captured on 6.5 µm microbeads with anti-CD63 capture antibodies	IACUC of Hanyang University (HYIACUC 2017-0050A) and Sungkyunkwan University (SKKUIACUC2021-04-38-1, SKKUIACUC2021-05-02-1)	Student's two-tailed *t*-test; one-way ANOVA + Tukey's *post-hoc*; GraphPad Prism 8; p> 0.05	[12]
1.5×10[6] SMSCs inoculated in 15 cm dish for 24 h until 70 to 80 % confluence	LPS preconditioning (LPS-pre EVs arm)	Complete culture medium with IL-1? (10 ng/mL) for in vitro stimulus; specific EV production medium NR in extracted text	NR	Differential centrifugation + ultrafiltration	Centrifugation 30 min at 10,000*g* to remove tiny fragments; supernatant filtered through 0.22 µm filter; further centrifugation steps deferred to a referenced protocol	NR specific UC	NR	Not used	TEM, NTA, WB (*per* Table 1)	NR (TEM used; specific model NR)	NR specific in extracted text	Not performed	Anti-CD9 (1:2000; Abcam ab92726); anti-CD63 (1:2000; Abcam ab182975); anti-Alix; anti-TSG101; collagenase type II 2 mg/mL (Gibco) for cartilage digestion	Research ethics committee of The First Affiliated Hospital	One-way ANOVA; **p*<0.05; ***p* <0.01; ****p*<0.001	[13]

Cell passage at EV harvest	EV priming / genetic modification	EV production medium (composition)	Conditioning time, h	EV Isolation method	Isolation protocol - RCF / time / steps	Isolation equipment (rotor, ultracentrifuge model)	Isolation temperature	Commercial kit (catalog/manufacturer if used)	Characterisation techniques (overview)	TEM Instrument (model + accelerating voltage)	NTA / particle sizer (model + manufacturer)	DLS / Zetasizer instrument	Western Blot markers + antibodies (clone, manufacturer, catalog)	Ethics approval / regulatory ID	Statistical methods	Ref.
NR	Mechanical stimulation by Rotary Cell Culture System (RCCS); also lncRNA H19 modulation; UC-MSC-7530 reference cell line	Exosome-free conditioned medium	NR	Ultracentrifugation	Through 0.22 µm filter, then centrifuged at 110,000*g* for 70 min; suspension recentrifuged at 110,000*g* for 70 min	NR specific UC	All operations performed at low temperature (specific °C NR)	Not used	TEM, NTA, FC (*per* Table 1)	NR (TEM used; method described)	ZetaView instrument (NTA)	Not performed	EV markers: CD63, CD81, TSG101 (positive); calnexin (negative); MSC markers: CD105, CD73, CD90 (positive); CD31 (negative)	NR specific in extracted text	Unpaired Student's *t*-test; one-way ANOVA; GraphPad Prism v8.0	[14]
NR	Lentiviral overexpression of miR-140-5p (hUSC-140-Exos)	NR specific in extracted text	NR	Ultracentrifugation	Per PDF: ultracentrifugation (specific RCF/time NR in extracted text)	NR	NR	Not used	TEM, NTA, WB (per Table 1)	NR in extracted text	NR in extracted text	Not performed	NR specific markers in extracted text	Ethics committee for biomedical research at West China Hospital, Sichuan University	Independent t-tests; Mann-Whitney U; SPSS v22.0	[15]
NR	NR	NR	NR	Ultracentrifugation (*per* Table 1)	NR	NR	NR	Not used *per* Table 1	I Table 1: characterization listed as blank	NR	NR	NR	NR	NR	NR	[16]
NR	None	NR specific	48 h conditioning of conditional medium	Sequential ultracentrifugation	Conditioned medium pre-cleared; supernatant transferred to ultracentrifuge tube and centrifuged at 100,000*g* for 70 min to pellet EVs	NR specific UC model	NR (4 °C implied for UC steps)	Not used	TEM, WB, DLS (*per* Table 1)	Hitachi HT7700 TEM at 100 kV accelerating voltage	Not performed (DLS used)	DLS performed; specific instrument NR	Anti-CD9 (GTX66709, GeneTex; 1:1000); anti-CD81 (GTX31381; 1:1000); anti-TSG101 (GTX70255; 1:1000); anti-CD63 (EXOAB-CD63A-1, System Biosciences; 1:1000); anti-Collagen II (Abcam, 1:1000); also anti-p21	NR specifically in extracted summary (Stem Cell Research guidelines in Taiwan compliant)	GraphPad Prism software 8.0.1; **p* < 0.05; ***p* < 0.01	[17]
NR	None (also evaluated antagomiR delivery)	DMEM/F12 (Gibco) + 20 % FBS + 1 % NEAA initially; production medium NR specific	NR	Differential centrifugation	Sequential differential centrifugation: low-speed / 100,000*g* for 90 min / resuspend in 20 mL PBS / re-centrifuge at 100,000*g*; isolation protocol previously detailed (referenced)	NR specific UC	NR	Not used	TEM, NTA, WB (per Table 1)	Tecnai 12 transmission electron microscope (Philips, Netherlands)	NR specific in extracted text	Not performed	Anti-CD9 (1:2000; Abcam ab223052); anti-CD63 (1:2000; Abcam ab134045); anti-Alix; anti-TSG101 (1:2000; Proteintech 14497-1-AP)	Animal Research Committee of Nanjing Medical University (IACUC-2112016)	GraphPad Prism 8.0; SPSS 26.0; independent-sample *t*-test; multiple comparisons	[18]
NR	None	NR specific in extracted text	NR	Sequential ultracentrifugation + ultrafiltration (300-kDa)	Excess dye aggregates removed using 300-kDa ultrafiltration; SEC purification (Echo Biotech, Beijing)	300-kDa ultrafiltration; SEC column (Echo Biotech, Beijing)	NR specific	SEC column (Echo Biotech, Beijing)	TEM, AFM, SEM (FEI QUANTA 450), FC (*per* Table 1)	NR specifically (TEM used)	Not performed (Table 1 lists FC instead)	Not performed	FITC-anti-human CD63 (BD); FITC-anti-human CD9 (BD); FITC-anti-human CD81 (BD); CD63 (Santa Cruz)	Institutional Animal Ethics Committee of Fujian Medical University (FJMU IACUC 2020–0052)	One-way ANOVA + nonparametric tests; SPSS 20.0; *p* < 0.05	[19]

CC BY

Cell passage at EV harvest	EV priming / genetic modification	EV production medium (composition)	Condition-ing time, h	EV Isolation method	Isolation protocol - RCF / time / steps	Isolation equipment (rotor, ultra-centrifuge model)	Isolation tempe-rature	Commercial kit (catalog/manufacturer if used)	Characterisation techniques (overview)	TEM Instrument (model + accelerating voltage)	NTA / particle sizer (model + manufacturer)	DLS / Zetasizer instrument	Western Blot markers + antibodies (clone, manufacturer, catalog)	Ethics approval / regulatory ID	Statistical methods	Ref.
NR	None (also miR-199a-3p delivery arm)	DMEM (Gibco, USA) + 5 % CO_2 at 37 °C; specific exosome production medium NR	NR	Sequential differential centrifugation	2,000*g* for 20 min, 10,000*g* for 30 min to eliminate dead cells/debris; then ultracentrifugation (specific final RCF/time NR in extracted text)	NR specific	NR	Not used	TEM, NTA (*per* Table 1)	NR specific	NR specific	Not performed	NR specific in extracted text	Approval Number: TJAA07622701, Tongji University	GraphPad Prism 8.0 (GraphPad Software, Inc., La Jolla, CA, USA); $p < 0.05$	[20]
NR	Hypoxic preconditioning (10 % O_2 - inflammaging study); compared with normoxic 20 % O_2	NR specific composition	NR	Ultracentrifugation	ADSC-Exos isolated from CM by ultracentrifugation (specific RCF/time/temp NR in extracted text)	NR specific UC model	NR	Not used	TEM, NTA (*per* Table 1); WB	NR specific (TEM used)	NR specific	Not performed	Anti-CD9 (Cat. 60232-1-Ig, Proteintech, Rosemont, IL); anti-CD63; anti-CD81; anti-ALIX; anti-TSG101	Animal Care and Use Committee of Kaohsiung Medical University (IACUC number 109242); ethical committee at the Kaohsiung Medical University for human samples	Mean ± SEM, *n* = 6 replicates; $p < 0.05$; software NR specific	[21]
NR	Engineered: CLS-PEG-MAL and CLS-PEG-CAP modifications (chondrocyte-targeting and controlled release system); also therapeutic miRNA loading	NR specific in extracted text	NR	Gradient centrifugation	Gradient centrifugation method (specific densities/RCF/time NR in extracted summary)	NR specific	NR	Not used	TEM, NTA, WB (per Table 1: blank but text confirms)	TEM used; specific model NR in extracted text	NTA used; specific model NR in extracted text	Not performed	EV markers CD63 and TSG101 (positive)	West China Hospital Ethics Committee, Sichuan University (Approval Number: 2022200)	*p<0.05; **p<0.01; ***p<0.001 mean ± SD, *n* = 3	[22]
NR	None	NR specific composition	NR	Ultracentrifugation	Ultracentrifugation of DPSC conditioned medium (specific RCF/time NR in extracted text; matches Table 1: Sequential ultra-centrifugation')	NR specific	NR	Not used	TEM, NTA, WB (*per* Table 1)	NR specific	NR specific	Not performed	Exosome markers CD9 and CD81 (positive); MSC markers CD105, CD29, CD90, CD73 (positive); CD34, CD45 (negative)	Ethics Committee of the Peking University School of Stomatology (PKUSSIRB-201311103); Peking University Animal Ethics Committee	Mean ± SD, *n* = 5; *p<0.05, **p<0.01, ***p<0.001	[23]
Not applicable (direct tissue isolation, not cell culture)	None	NR (tissue-derived isolation)	Tissue at room temperature for 2 h initial; sterilized through 0.22 µm filters	Sequential centrifugation + purification	Supernatant underwent sequential centrifugations at 3,000×g, 10,000×g, and 100,000×g; pellets then purified	NR specific UC	NR (RT initial, low T for UC implied)	Not used (sequential UC)	NTA, WB (*per* Table 1); MicroBCA (Thermo Fisher)	NR specific (TEM not in Table 1 char list)	NR specific	Not performed	Anti-CD9 (Cat# 10626D, Thermo Fisher Scientific); anti-CD63 (Cat# 10628D); anti-CD81 (Cat#); MicroBCA protein assay kit (Thermo Fisher)	IACUC of Pharmaseed (Ness-Ziona, Israel)	GraphPad Prism 9.3.1; *t*-test; one-way and two-way ANOVA with Tukey's multiple comparisons	[24]

Cell passage at EV harvest	EV priming / genetic modification	EV production medium (composition)	Conditioning time, h	EV Isolation method	Isolation protocol - RCF / time / steps	Isolation equipment (rotor, ultracentrifuge model)	Isolation temperature	Commercial kit (catalog/manufacturer if used)	Characterisation techniques (overview)	TEM Instrument (model + accelerating voltage)	NTA / particle sizer (model + manufacturer)	DLS / Zetasizer instrument	Western Blot markers + antibodies (clone, manufacturer, catalog)	Ethics approval / regulatory ID	Statistical methods	Ref.
NR	None	NR specific	NR	NR specifically (Table 1: NR; text mentions 0.22 µm filtration of supernatant + 10,000*g* for 5 min)	Supernatant collected after 10,000*g* for 5 min; passed through 0.22 µm filter; used for IA injection (Note: this describes UCMSC SUPERNATANT, not classical exosome isolation - paper investigates UCMSC supernatant rather than purified Exos)	NR	NR	Not used	*Per* Table 1: blank; text describes UCMSC supernatant rather than EV preparation	NR	NR	Not performed	NR specific markers in extracted text	Animal Care and Use Committee, Zhejiang University (No. 18021); ICLAS guidelines	Unpaired two-tailed *t*-test after Shapiro-Wilk normality and Brown-Forsythe equal variance; ANOVA; $*p < 0.05$	[25]
NR	None	NR specific	NR	Tangential flow filtration (TFF; 50× concentration)	Conditioned medium size-fractionated and concentrated 50× by TFF using a membrane with specific molecular weight cut-off; filtered through 0.22 µm filter (Merck)	TFF system (specific make NR)	NR	Not used	TEM, NTA, FC (*per* Table 1); BCA	NR specific (TEM used)	NanoSight LM10-12	Not performed	Exosomal markers CD81, CD9, CD63 analysed by flow cytometry; anti-Collagen I antibody for IHC; MSC markers CD73, CD90, CD105 (positive); BCA protein assay kit (Boster Biological Technology Co.)	NR specific in extracted summary	GraphPad Prism v5.01; paired *t*-test analysis	[26]
NR (cells seeded at 6,250 cells/cm^2 *per* text)	Standardized manufacturing process	NR specific composition (GMP-compatible referenced)	NR	Serial ultracentrifugation	Pre-clearing / 100,000*g* for 1 h at 4 °C / SN discarded ? resuspend / 100,000*g* for 1 h at 4 °C; serial ultracentrifugation (Thermo Electron LED GmbH)	Thermo Electron LED GmbH ultracentrifuge	4 °C	Not used	TEM, NTA, FC (*per* Table 1); proteomics	TEM at the Advanced Microscopy Facility UMA-UC (PUC); specific model NR	NR specific	Not performed	Bead-based FC: CD63, CD81, CD9 (sEV surface markers); CD44, CD90 (MSC origin markers; BioLegend); Syntenin-1 (sEV endosomal origin marker); Flotillin-1; analysed on FlowJo software V10	Scientific Ethics Committee of Universidad de los Andes (IACUC approval certificate)	GraphPad Prism (V10.2.0); Student's *t*-test, one-way ANOVA	[27]
NR	None	NR specific	NR	Ultracentrifugation	*Per* text: ultra-centrifugation method (specific RCF/time NR in extracted summary; Beckman Coulter Brea, CA referenced for 1 centrifugation step)	Beckman Coulter (Brea, CA, USA) specific model NR	NR	Not used	TEM, ZS (Zetasizer), WB (*per* Table 1)	NR specific	Not performed (ZS used instead)	Zetasizer (ZS) specific model NR	EV markers: CD63 (52.5% positive), CD81; MSC markers CD105, CD90, CD44, CD73 (positive)	Ethics committee approved (NR specific)	$*p<0.05$; $**p<0.01$; $***p<0.001$ noted	[28]

(cc) BY

Cell passage at EV harvest	EV priming / genetic modification	EV production medium (composition)	Conditioning time, h	EV Isolation method	Isolation protocol - RCF / time / steps	Isolation equipment (rotor, ultracentrifuge model)	Isolation temperature	Commercial kit (catalog/manufacturer if used)	Characterisation techniques (overview)	TEM Instrument (model + accelerating voltage)	NTA / particle sizer (model + manufacturer)	DLS / Zetasizer instrument	Western Blot markers + antibodies (clone, manufacturer, catalog)	Ethics approval / regulatory ID	Statistical methods	Ref.
Passages 10–20	TGF-?3 (10 ng *per* mL, 24 h) preconditioning in proliferative medium prior to EV production	DMEM + 100 µg *per* mL penicillin/ streptomycin + 2 mmol *per* mL glutamine + 3 % EV-depleted FCS (FCS depleted by ultracentrifugation at 100,000*g* overnight, 4 °C)	48 h	Differential ultracentrifugetion (separate MP and Exo fractions)	Conditioned medium / 300*g*, 10 min (cell removal) / 2,500*g*, 25 min (debris/apoptotic body removal). MP fraction: pellet at 18,000*g* for 1 h in polyallomer tubes, resuspended in PBS, re-spun at 18,000*g*. Exo fraction: MP-depleted supernatant filtered through 0.22 µm membrane, then 100,000*g* for 2 h ×2 (second wash in PBS). Final pellets resuspended in 100 µL PBS, freshly used.	NR (rotor and ultracentrifuge model not specified)	4 °C (for EV-depleted FCS preparation; in-process temperature NR for main UC steps)	Not used (differential ultracentrifugation only)	BCA (Bradford colorimetric assay) for protein quantification; NTA; DLS; TEM; flow cytometry on bead-coated EVs	NR (TEM used; model/voltage not specified in main text)	NanoSight LM10-12 (Malvern)	Dynamic light scattering (manufacturer NR)	Bead-based flow cytometry markers (not WB): CD9 (clone MZ3, Miltenyi Biotec), CD29 (clone Ha2/5, BD Biosciences), CD44 (clone IM7, BD Biosciences), CD81 (clone EAT2, Miltenyi Biotec), SCA-1 (clone D7, BD Biosciences); EV proteins (1 µg) coated onto 4 µm aldehyde/sulfate latex beads at 4 °C overnight, blocked with 100 mM glycine	Ethical Committee for animal experimentation of the Languedoc-Roussillon (Approval 5349-20160509181-98875); French Ministry for Education, Higher Education and Research	Mann–Whitney for in vitro nonparametric data; Student's *t*-test for *in vivo* (*n* = 15 *per* group); GraphPad Prism 6; $p < 0.05$ significant	[29]
NR (BM-MSCs from rat femur/tibia; phenotyped CD90+/CD11-/CD45-)	None	Low-glucose DMEM + 10 % exosome-free FBS (Gibco, USA)	48 h	Differential ultracentrifugation	Conditioned medium centrifuged at 110,000*g* for 1 h at 4 °C; pellet washed and resuspended in PBS; second 110,000*g* for 1 h at 4 °C.	Type 45 Ti rotor (Beckman Coulter, USA)	4 °C	Not used	TEM, DLS (Zetasizer), Western blot for CD63, TSG101, Flotillin-1	HITACHI H-7000FA (Japan), 100 kV accelerating voltage	Not performed (DLS used instead)	Zetasizer Nano (Malvern, UK)	Anti-CD63 (ProteinTech, USA); anti-TSG101 (Abcam, UK); anti-Flotillin-1 (Abcam, UK); secondary antibodies (Abcam, UK); detection on Tanon 5200 (Tanon Science & Technology, China)	Animal Care and Use Committee of Sun Yat-sen University	NR in extracted methods text	[30]
NR (refers to prior protocol [18])	Adenovirus-mediated Runx2 overexpression (Ad-Runx2; Shanghai Jikai Biotech) for R-BMSC arm	NR (conditioned medium of BMSCs and Runx2-transfected BMSCs)	NR	NR (deferred to reference [20]; only protein extraction kit specified for downstream WB)	NR in main methods (only Total Exosome Protein Isolation Kit referenced for protein extraction step, not isolation)	NR	NR	Total Exosome Protein Isolation Kit (Invitrogen, USA) used for downstream protein extraction, not vesicle isolation	NTA, TEM, Western blot for CD63, CD81, TSG101	NR (TEM used; model/voltage not specified)	NR (manufacturer not specified)	Not performed	Anti-COL-II, anti-SOX9, anti-Runx2 (Abcam, Cambridge, MA, USA); anti-YAP, anti-CD63, anti-CD81, anti-TSG101 (System Biosciences, Palo Alto, CA, USA); anti-CTGF, anti-Ankrd1, anti-GAPDH (manufacturer in original)	Ethics Committee of Wuhan Third Hospital, Tongren Hospital of Wuhan University; Lenience No. SCXK Hubei 2019-0011 (rabbit supplier)	Mixed model repeated measures (MMRM); histological evaluations compared between groups	[31]

Cell passage at EV harvest	EV priming / genetic modification	EV production medium (composition)	Conditioning time, h	EV Isolation method	Isolation protocol - RCF / time / steps	Isolation equipment (rotor, ultracentrifuge model)	Isolation temperature	Commercial kit (catalog/manufacturer if used)	Characterisation techniques (overview)	TEM Instrument (model + accelerating voltage)	NTA / particle sizer (model + manufacturer)	DLS / Zetasizer instrument	Western Blot markers + antibodies (clone, manufacturer, catalog)	Ethics approval / regulatory ID	Statistical methods	Ref.
Passage 2	Lipofectamine 2000 transfection of miR-9-5p mimics, miR-9-5p inhibitors, or scrambled NCs (100 pmol in 250 µL serum-free medium + 10 µL Lipofectamine 2000 in 250 µL serum-free medium) into BMSCs prior to EV isolation; 4 h transfection + 48 h culture	DMEM + exosome-free FBS	24 h	Sequential / differential ultracentrifugation	Sequential centrifugation: 300*g* (5 min), 2,000×g (10 min), 10,000*g* (35 min); supernatant filtered through 0.22 µm membrane (Merck Millipore, Tullagreen, Ireland); ultracentrifugation at 100,000*g* for 2 h ×2; final resuspension in 50to100 µL PBS; stored at -80 °C.	Beckman Optima L-90K ultracentrifuge with SW-32 Ti rotor; low-speed steps on Beckman Coulter Allegra X-15R	4 °C (all UC steps)	Not used	Western blot only (CD9, TSG101, calnexin, CD63); no TEM/NTA/DLS reported	Not performed	Not performed	Not performed	Anti-CD9, anti-TSG101, anti-calnexin, anti-CD63 (all 1:1000); HRP-IgG secondary (1:1000); BCA for protein; ECL detection	Animal Ethics Committee of First Hospital of China Medical University; NIH Guide compliant	NR in extracted methods text	[32]
Passages 2–5	None	MEM (Thermo Fisher Scientific, Waltham, MA) + 20 % FBS, then exosome-free serum for production phase	NR (described as sequential centrifugation of conditioned medium)	Sequential ultracentrifugation (per ref 25)	BMSCs in exosome-free serum; conditioned medium collected by sequential centrifugation; exosomes pelleted, resuspended in PBS, stored at -80 °C. Specific RCF/time values referenced to prior protocol [25] rather than restated.	NR (specific UC model/rotor not stated)	NR	Not used	TEM (described as 'SEM' in figure legend — likely typographical), BCA, Western blot for CD9, CD63, TSG101 (negative for GM130)	NR (TEM used; model not specified)	Not performed	Not performed	Anti-CD9, anti-CD63, anti-TSG101 (manufacturer NR); BCA: Pierce BCA Protein Assay Kit (Thermo Fisher Scientific)	Animal Investigation Committee of the Institute of Biomedical Sciences, Central South University (No: 2019-S036)	SPSS 22.0; two-way ANOVA for behavioral data; unpaired *t*-test and one-way ANOVA with Tukey-Kramer *post-hoc* for other comparisons; p<0.05	[33]
After density gradient centrifugation, expanded with medium changed every 2 days; passaged at 80 to 90 % confluence at 1:2 ratio (specific passage at EV harvest NR)	None	MEM (HyClone, Logan, UT)+10 % FBS (Gibco, CA)+1 % penicillin/streptomycin (HyClone) / switched to FBS without exosomes for production phase	48 h	Differential ultracentrifugation	10,000*g* for 30 min (clearance of cell debris) / 100,000*g* for 120 min (first ultracentrifugation) / resuspend pellet / 0.22 µm sterile filter (Millipore, Boston, MA) to remove larger EVs / second 100,000*g* for 120 min / resuspended in PBS (Solarbio, Beijing, China); stored at ?80 °C.	Ultracentrifuge : HITACHI Limited (Tokyo, Japan)	NR (not stated for centrifugation steps)	Not used	TEM, NTA (nanoparticle sizer), Western blot for CD81, CD63, TSG101; BCA	HITACHI Limited (Tokyo, Japan); accelerating voltage NR	Nanoparticle sizer (Malvern, Malvern, UK); model not specified	Not performed	Anti-CD81 (Santa Cruz, USA); anti-CD63 (Santa Cruz, CA, USA); anti-TSG101 (Abcam, Cambridge, MA); BCA: Beyotime, Shanghai, China; PVDF membrane: Whatman, Maidstone, Kent, UK; chemiluminescent imaging: GE, Boston, USA	Animal Research Ethics Committee of Jinzhou Medical University (2022025)	GraphPad Prism 8.0; Student's *t*-test (2 groups); one-way ANOVA with Tukey *post-hoc* (?3 groups); $p < 0.05$	[34]

(cc) BY

Cell passage at EV harvest	EV priming / genetic modification	EV production medium (composition)	Condition-ing time, h	EV Isolation method	Isolation protocol - RCF / time / steps	Isolation equipment (rotor, ultra-centrifuge model)	Isolation tempe-rature	Commercial kit (catalog/manufacturer if used)	Characterisation techniques (overview)	TEM Instrument (model + accelerating voltage)	NTA / particle sizer (model + manufacturer)	DLS / Zetasizer instrument	Western Blot markers + antibodies (clone, manufacturer, catalog)	Ethics approval / regulatory ID	Statistical methods	Ref.
Generations 3-5	TUC339-OV vector arm (lncRNA TUC339 over-expression) in addition to standard BMSC-Exo arm	DMEM complete medium + 10 % FBS	NR (passaged at 80–90% confluence; conditioned medium collected at this stage)	ExoQuick Extraction Kit + ultrafiltration centrifugation	Cell culture fluid centrifuged at 2,000*g* for 10 min (debris removal) / filtered through 0.45 µm sterile membrane / 2,000*g* for 10 min / transferred to ultrafiltration centrifuge tube / 100,000*g* for 15 min / BMSCs-exo extracted per Exo Quick Extraction Kit instructions.	NR (ultrafiltration tube; UC model not specified)	NR	ExoQuick Extraction Kit (manufacturer not stated; product attributable to System Biosciences)	TEM with phosphotungstic acid (pH 6.8) negative staining; NTA; Western blot for TSG101, CD63, CD81; BCA	NR (TEM used; model not specified)	Malvern Panalytical (Netherlands); software: Version 2.3 Build 0034	Not performed	Anti-TSG101, anti-CD63, anti-CD81 (manufacturer NR); BCA: Abcam, USA; Trizol: Thermo Fisher; PCR kits: Thermo Fisher	NR	SPSS 21; independent-samples t-tests; ANOVA with LSD post-hoc; ROC analysis; Pearson correlation; $p< 0.05$	[35]
Subculture at ~80 % confluence; specific passage at EV harvest NR	miR-326 mimics overexpression (Lipofectamine 2000; GenePharma) for one arm	MEM (Gibco, USA) + 10 % FBS+100 U/mL penicillin (Solarbio, China)+10 mg mL^{-1} streptomycin (Solar-bio); switched to exosome-free media for production	NR (production medium and culture described, but specific conditioning duration not stated in extracted text)	ExoQuick-TC™ kit (commercial polymer precipitation)	Media + PBS-washed cell wash collected and centrifuged 5 min, then supernatant centrifuged 20 min; exosomes isolated with the ExoQuick-TC™ system per protocol.	NR	NR	ExoQuick-TC™ (System Bioscience, USA)	NTA, TEM (uranyl acetate negative staining), Western blot for CD9, CD63, CD81; BCA	NR (TEM used; model NR; copper grid + 2 % uranyl acetate)	Nanosizer™ (Malvern Instru-ments, UK); analysed with Zetasizer software	Not performed	Anti-CD9 (ab92726, Abcam); anti-CD63 (ab217345, Abcam); anti-CD81 (ab109201, Abcam); anti-HDAC3 (ab32369, Abcam); anti-STAT1 (ab230428, Abcam); anti-NF-?B p65 (8242, Cell Signaling); anti-NLRP3 (13158S, CST); anti-ASC (67824T, CST); anti-GSDMD (ab209845, Abcam); anti-Caspase-1 (Nbp2-15713, Novus); anti-IL-1? (12242, CST); anti-IL-18 (57058, CST); anti-GAPDH (ab8245, Abcam); BCA: Thermo Fisher	Animal Ethics Committee of Anhui Medical University; NIH Guide compliant	GraphPad Prism 8.0; one-way ANOVA and Student's *t*-test; $p < 0.05$	[36]

Cell passage at EV harvest	EV priming / genetic modification	EV production medium (composition)	Conditioning time, h	EV Isolation method	Isolation protocol - RCF / time / steps	Isolation equipment (rotor, ultracentrifuge model)	Isolation temperature	Commercial kit (catalog/manufacturer if used)	Characterisation techniques (overview)	TEM Instrument (model + accelerating voltage)	NTA / particle sizer (model + manufacturer)	DLS / Zetasizer instrument	Western Blot markers + antibodies (clone, manufacturer, catalog)	Ethics approval / regulatory ID	Statistical methods	Ref.
Passage 5 used for surface-marker characterization; passaging at 80 to 90 % confluence	None	Serum-free stem cell medium (Gibco, USA) for production; baseline DMEM/F-12 (Gibco) + 10 % FBS (Gibco) + 1 % pen/strep (Sigma)	48 h	Sequential ultracentrifugation + sucrose/D2O cushion + ultrafiltration	300*g*, 10 min / 2,000*g*, 10 min at 4 °C / 0.22 µm filtration / Amicon Ultra-15 centrifugal filter (Burlington, USA) at 4,000*g* at 4 °C until volume reduced to 200 µL / washed twice with PBS by ultrafiltration / loaded onto 30 % sucrose/D?O pad in Ultra-Clear™ tube (Beckman Coulter) / 100,000*g* for 60 min at 4 °C in Optima L-100 XP Ultracentrifuge (Beckman Coulter); exosomes recovered with 18 g needle, diluted with PBS, re-concentrated through Amicon filter to 200 µL final volume; stored at ?80 °C.	Beckman Coulter Optima L-100 XP Ultracentrifuge; Ultra-Clear™ tubes (Beckman); Amicon Ultra-15 (Burlington, USA)	4 °C (all steps)	Not used	NTA, TEM, Western blot for CD81, CD9, CD63	NR (TEM used; model not specified)	Nanosight Tracking Analysis (model NR)	Not performed	Anti-CD81, anti-CD9, anti-CD63 (Abcam, USA); also anti-Collagen X, anti-Collagen II, anti-Runx2, anti-SOX9, anti-GAPDH (Abcam, USA); HRP-secondary (Abcam); ECL: Beyotime	Animal Care and Use Committee of Nanjing Medical University	SPSS 20.0; Student's *t*-test; one-way and two-way ANOVA; $p < 0.05$	[37]
Passage 3	Hypoxic preconditioning: 1 % O_2+5 % CO_2 + balanced N_2 (Hypo-ADSC-Exo arm); compared to normoxic ADSC-Exo	Complete DMEM + exosome-free FBS	NR (cultured under hypoxic vs. normoxic conditions; specific harvest time not stated in extracted text)	Commercial precipitation kit (ExoQuick-TC)	Per manufacturer's protocol of ExoQuick-TC kit applied to conditioned medium	NR	NR	ExoQuick-TC (System Biosciences, Mountain View, CA)	NTA, TEM, Western blot for CD63, CD81, TSG101	HITACHI H-7000FA (Japan); accelerating voltage NR	ZetaView (Germany); model not specified	Not performed	Anti-CD63, anti-CD81, anti-TSG101 (manufacturers NR in extracted text)	Ethics Committee of A University (No: 2020sydw0374); NIH Guide compliant	SPSS 25.0; two-way ANOVA with Tukey *post-hoc* (behaveoural); one-way ANOVA with Tukey (multi-group); unpaired t-test (two groups); $p < 0.05$	[38]

(cc) BY

Table S2. Reporting quality of included studies based on MISEV Guidelines. Each cell scored from data extracted into Table S1 (the comprehensive methodological matrix). Symbols: ✓ = adequately reported per MISEV 2023; ◐ = partially reported; ✗ = not reported. See Footnotes & Rubric sheet for the explicit scoring criteria applied *per* column

Study ID	Isolation method	Characteriazation method	Size distribution	Surface marker analysis	Functional testing	Reporting clarity	Statistical methods	Adherence status	Ref
SD1	✓	✓	✓	✓	✓	✓	◐	Substantial adherence	[1]
SD2	✗	✓	◐	✓	◐	✗	✓	Partial adherence	[2]
SD3	◐	◐	✗	✓	◐	✗	◐	Limited adherence	[3]
SD4	✓	✓	✗	✓	✓	◐	✗	Partial adherence	[4]
SD5	✓	✓	◐	✗	◐	◐	✗	Limited adherence	[5]
SD6	✓	◐	✗	✓	◐	◐	◐	Limited adherence	[6]
SD7	◐	✓	✗	✗	✗	✗	✗	Limited adherence	[7]
SD8	✓	✓	✗	✓	◐	◐	✗	Partial adherence	[8]
SD9	✓	◐	✗	✓	✓	◐	◐	Partial adherence	[9]
SD10	✓	✓	◐	✓	◐	◐	◐	Partial adherence	[10]
SD11	✓	✓	◐	✓	✗	◐	◐	Partial adherence	[11]
SD12	✓	✓	◐	✓	◐	◐	✓	Partial adherence	[12]
SD13	◐	✓	✗	✓	✓	◐	◐	Partial adherence	[13]
SD14	✓	✓	◐	✓	✗	◐	◐	Partial adherence	[14]
SD15	✓	✓	✗	✗	◐	✗	◐	Limited adherence	[15]
SD16	◐	✗	✗	✗	◐	✗	✗	Limited adherence	[16]
SD17	✓	✓	◐	✓	◐	◐	◐	Partial adherence	[17]
SD18	◐	✓	✗	✓	◐	◐	◐	Limited adherence	[18]
SD19	◐	✓	◐	✓	✓	◐	✓	Partial adherence	[19]
SD20	✓	◐	✗	✗	◐	◐	◐	Limited adherence	[20]
SD21	✓	✓	✗	✓	✓	✗	◐	Partial adherence	[21]
SD22	◐	✓	✓	✓	◐	✗	◐	Partial adherence	[22]
SD23	✓	✓	✗	✓	✓	✗	◐	Partial adherence	[23]
SD24	✓	◐	✗	✓	✓	◐	◐	Partial adherence	[24]
SD25	✗	✗	✗	✗	✓	◐	◐	Limited adherence	[25]
SD26	✓	✓	◐	✓	◐	◐	◐	Partial adherence	[26]
SD27	✓	✓	✗	✓	◐	✓	◐	Partial adherence	[27]
SD28	◐	✓	◐	✓	✗	◐	◐	Limited adherence	[28]
SD29	✓	✓	◐	✓	✓	◐	✓	Substantial adherence	[29]
SD30	✓	✓	◐	✓	✓	✓	✗	Substantial adherence	[30]
SD31	✗	✓	✗	✓	◐	◐	✗	Limited adherence	[31]
SD32	✓	✓	◐	✓	✓	✓	✗	Substantial adherence	[32]
SD33	◐	✓	✓	✓	◐	✗	✓	Partial adherence	[33]
SD34	✓	✓	◐	✓	◐	◐	✓	Partial adherence	[34]
SD35	✓	✓	◐	✓	◐	◐	✓	Partial adherence	[35]
SD36	✓	✓	◐	✓	✓	✗	✓	Substantial adherence	[36]
SD37	✓	✓	◐	✓	◐	✓	✓	Substantial adherence	[37]
SD38	✓	✓	✓	✓	✓	◐	✓	Substantial adherence	[38]
	✓ 26/38 (68%)<br>◐ 9/38; ✗ 3/38	✓ 31/38 (82%)<br>◐ 5/38; ✗ 2/38	✓ 4/38 (11%)<br>◐ 17/38; ✗ 17/38	✓ 32/38 (84%)<br>◐ 0/38; ✗ 6/38	✓ 14/38 (37%)<br>◐ 20/38; ✗ 4/38	✓ 5/38 (13%)<br>◐ 23/38; ✗ 10/38	✓ 10/38 (26%)<br>◐ 20/38; ✗ 8/38	Complete: 0; Substantial: 7<br>Partial: 20; Limited: 11	

Table S3. Dosage information of all included studies

Donor species	Delivery route	Concentration, particles mL^{-1}	Protein concentration, µg mL^{-1}	Volume injected, µL	Number of injection	Group	Dose *per* injection	Followup days	Ref
Human	IA	10^{10}	NR	8	3	Multiple	$8.0×10^7$	21	[1]
Human	IA	10^{11}	NR	100	4	Multiple	10^{10}	84	[2]
Human	IA	NR	NR	5	9 (every 3 days × 4 weeks)	Multiple	NR	28	[3]
Human	IA	10^{10}	NR	10	8 (twice weekly ×4	Multiple	10^8 particles	28	[4]
		10^{10}	NR	10	12 (twice weekly ×6)	Multiple	10^8 particles	42	
		10^{10}	NR	10	8 (twice weekly ×4)	Multiple	10^8 particles	28	
		10^{10}	NR	10	8 (twice weekly ×4)	Multiple	10^8 particles	28	
Human	IA	$3.33×10^9$	NR	30	3 (weekly ×3)	Multiple	10^8 particles	28	[5]
		$3.33×10^9$	NR	30	11 (≈ twice weekly ×40 days)	Multiple	10^8 particles	56	
Human	IA	NR	NR	10	3 (days 7, 14, 21)	Multiple	NR	21	[6]
		NR	NR	10	3 (days 7, 14, 21)	Multiple		21	
Human	IA	NR	NR	100	Weekly at week 1 after OA induction	Multiple	NR	5	[7]
Human	IA	NR	NR	5	9 (every 3 days × 4 wks)	Multiple	NR	84	[8]
		NR	NR	5	9 (every 3 days × 4 wks)	Multiple	NR	84	
Human	IA	NR	30	200	4 (weekly ×4)	Multiple	30 µg	63	[9]
Human	IA	NR	100	25	3 (days 15, 22, 29)	Multiple	2.5 µg	21	[10]
Human	IA	NR	100	25	3 (days 15, 22, 29)	Multiple	2.5 µg	21	
Human	IA	NR	100	100	8 (weekly ×8)	Multiple	100 µg	56	[11]
Human	IV	10^{10}	NR	100	1	Single	10^9 particles	<1 day (25 h max)	[12]
		10^{10} (calculated)	NR	100	1	Single	10^9 particles	<1 day (7 h max)	
Human	IA	10^{11}	NR	10	12 (twice weekly ×6)	Multiple	10^9 particles	42	[13]
		10^{11}	NR	10	12 (twice weekly ×6)	Multiple	10^9 particles	42	
Human	IA	NR	1000	100	4 (weekly ×4)	Multiple	100 µg	28	[14]
		NR	1000	100	8 (weekly ×8)	Multiple	100 µg	56	
		NR	1000	100	4 (weekly ×4)	Multiple	100 µg	28	
		NR	1000	100	8 (weekly ×8)	Multiple	100 µg	56	
Human	IA	10^{11}	NR	100	Weekly (≥4)	Multiple	10^{10} particles	28, 56… (every 4 weeks)	[15]
		10^{11}	NR	100	Weekly (≥4)	Multiple	10^{10} particles		
Human	IA	NR	NR	NR	4 (weekly ×4)	Multiple	NR	28	[16]
		NR	NR	NR	8 (weekly ×8)	Multiple	NR	56	
Human	IA	NR	NR	100	3 (weekly ×3)	Multiple	100 µg	21	[17]
Human	IA	10^{11}	NR	10	12 (twice weekly ×6)	Multiple	10^9 particles	42	[18]
		10^{11}	NR	10	12 (twice weekly ×6)	Multiple	10^9 particles	42	
		10^{11}	NR	10	12 (twice weekly ×6)	Multiple	10^9 particles	42	
Human	IA	NR	30	200	4 (weekly ×4)	Multiple	30 µg	63	[19]

CC BY

Donor species	Delivery route	Concentration, particles mL^{-1}	Protein concentration, µg mL^{-1}	Volume injected, µL	Number of injection	Group	Dose *per* injection	Followup days	Ref
Human	IA	2×10^{10}	NR	50	6 (weekly ×6)	Multiple	10^{9} particles	56	[20]
		2×10^{10}	NR	50	6 (weekly ×6)	Multiple	10^{9} particles	77	
		2×10^{10}	NR	10	4 (weekly ×4)	Multiple	10^{8} particles	70	
		2×10^{10}	NR	10	4 (weekly ×4)	Multiple	10^{8} particles	70	
		2×10^{10}	NR	10	4 (weekly ×4)	Multiple	10^{8} particles	70	
Human	IA	7.5×10^{9}	NR	40	7 (weekly ×7)	Multiple	3.0×10^{8} particles	49	[21]
Human	IA	NR	NR	NR	NR	NR	NR	NR	[22]
Human	IA	2×10^{10}	NR	10	1	Single	2×10^{8} particles	28	[23]
Human	IA	NR	20	30	1 (day 5)	Single	20 µg	39	[24]
		NR	20	30	3 (day 5,12,19)	Multiple	20 µg	25	[25]
Human	IP								[26]
Human	IA	4×10^{10}	NR	5	2 (days 7,14)	Multiple	2×10^{8} particles	28	[27]
Human	IA	10^{11}	NR	100	8 (2×/week ×4 weeks)	Multiple	0^{10} particles	56	[28]
C57BL/6 mice	IA	NR	0.25	5	1	single	0.25 µg	35	[29]
C57BL/6 mice	TV	NR	200	200	4	Multiple	200 µg	NR	[30]
C57BL/6 mice	IA	10^{10}	NR	10	9	Multiple	10^{8}	NR	[31]
SD Rats	IA	NR	NR	NR	NR	NR	NR	NR	[32]
SD Rats	IA	NR	40	100	5	Multiple	NR	42	[33]
SD Rats	IA	NR	40	100	5	Multiple	40 µg	42	[34]
		NR	40	100	5	Multiple	40 µg	42	
Rabbit	IA	NR	10	1000	5	Multiple	10 µg	56	[35]
		NR	10	1000	5	Multiple	10 µg	56	
C57BL/6 mice	IA	NR	500	NR	7	Multiple	NR	NR	[36]
		NR	NR	NR	7	Multiple	10^{9} PFU	NR	
C57BL/6 mice	IA	NR	NR	NR	NR	NR	NR	NR	[37]
		NR	NR	NR	NR	NR	NR	NR	
SD rats	IA	NR	100	50	1	single	100	42	[38]

Table S4. Meta-regression analysis of moderators in human-derived MSC-EV studies (OARSI score as outcome)

Moderator	Estimate	95% CI (lower - upper)	SE	*z*-value	p-value
Intercept	-7.76	-15.51 - -0.01	3.95	-1.96	0.050*
Dosage per injection 10^6	5.74	-6.69 - 18.17	6.34	0.9	0.366
Dosage per injection 10^8	5.57	-4.41 - 15.56	5.09	1.09	0.274
Dosage per injection 10^9	7.43	-6.46 - 21.32	7.09	1.05	0.294
Dosage per injection 2×10^8	4.2	-12.92 - 21.33	8.74	0.48	0.631
Dosage per injection 3×10^8	9.96	-0.96 - 20.87	5.57	1.79	0.074 †
Dosage per injection 5×10^5	2.05	-15.14 - 19.25	8.77	0.23	0.815
Dosage per injection 8×10^7	-2.23	-11.14 - 6.68	4.55	-0.49	0.623
Number of injections	0	-1.16 - 1.16	0.59	0	1
[38]	1.04	-17.98 - 20.05	9.70	0.11	0.915
[26]	10.47	0.76 - 20.18	4.95	2.11	0.035*
[16]	0.75	-7.12 - 8.61	4.01	0.19	0.853

Model statistics: Test of moderators: QM(12) = 13.45, p = 0.34; residual heterogeneity: QE(5) = 36.04, p <0.001; I^2 = 96.1% ; τ^2 = 17.43; significant at p <0.05; *borderline trend (p < 0.10)

Table S5. Outcome index

Outcome index	EV cargo analysis	Specific proteins & miRNAs	Ref
1.Macroscopic (ICRS score) 2. Histology (OARSI score) 3. IHC (Collagen I and II) 4. Chondrocyte migration and proliferation assays	x	NR	[1]
1. Histology (Safranin-O & Fast Green staining) 2. IHC (Collagen II, ACAN) 3. Chondrocyte counts 4. Cartilage degeneration scoring (OARSI score)	þ	Wnt5a and Wnt5b proteins enriched in SMSC-derived exosomes, miR-140-5p, miR-140-3p (low expression) and some other highly expressed miRNAs (miR-6090, miR-4516, miR-3960, miR-4459), but only miR-140-5p was functionally validated	[2]
1. Histology (Safranin O/Fast Green staining) 2. Cartilage degeneration scoring (OARSI score) 3. Immunohistochemistry (Collagen II, MMP13, ADAMTS5) 4. TUNEL staining (apoptosis in cartilage)	þ	Showed miR-92a-3p targets WNT5A to enhance chondrogenesis and protect cartilage	[3]
1. Histology (Safranin O/Fast Green staining) 2. Cartilage degeneration scoring (OARSI score) 3. Immunohistochemistry (Collagen II, MMP13, ADAMTS5, LC3, p62, mTOR) 4. Gait analysis (Catwalk system)	þ	miR-100-5p (functional, mTOR target); also let-7i-5p, miR-221-3p, miR-21-5p, miR-148a-3p, miR-199a-3p	[4]
1. Histology (Safranin O/Fast Green staining) 2. Cartilage degeneration scoring (OARSI score) 3. Immunohistochemistry (Collagen II, MMP13, ADAMTS5) 4. Micro-CT (subchondral bone changes)	þ	miR-21 (functional, PTEN/AKT pathway)	[5]
1. Histology (Safranin O/Fast Green staining) 2. Cartilage degeneration scoring (OARSI score)	þ	No miRNA, BMP4 pathway	[6]
1. Histology (OARSI score) 2. IHC (Collagen I, II and ACAN)	x	NR	[7]

(cc) BY

Outcome index	EV cargo analysis	Specific proteins & miRNAs	Ref
1. Histology (Safranin O/Fast Green, OARSI score) 2. IHC (Collagen II, MMP13, ADAMTS5)	þ	miR-135b	[8]
1. Histology (Safranin O/Fast Green, OARSI score) 2. IHC (Collagen II, MMP13, ADAMTS5)	þ	miR-140-3p (functional); other miRNAs listed but not validated	[9]
1. Histology (OARSI score) 2. IHC (Collagen I and II) 3.qPCR (collagen type I, Sox9, collagen type II, and aggrecan 4. Histology (H&E staining)	x	NR	[10]
1. Histology (Safranin O/Fast Green staining) 2. Cartilage degeneration scoring (OARSI score) 3. Immunohistochemistry (Collagen II, MMP13, ADAMTS5) 4. TUNEL staining (apoptosis in cartilage) 5. Micro-CT (subchondral bone changes)	þ	miR-26a-5p (validated, mechanistic)	[11]
1. Micro-CT outcomes (e.g., BMD, BV/TV, Tb.Th, Tb.N) 2. TRAP-positive osteoclast counts	þ	Osteoprotegerin (OPG), miR-21-5p (validated, targets Acvr2a), let-7b-5p (validated, reduces osteoclast gene expression)	[12]
1. Histology (Safranin O/Fast Green, OARSI score) 2. IHC (Collagen II, MMP13, ADAMTS5)	þ	let-7b	[13]
1. Histology (H&E, Safranin O/Fast Green, Toluidine Blue, IHC for Collagen II) 2. Macroscopic evaluation (ICRS) 3.MRI (T2 mapping) 4. Behavioral test (Von Frey, pain response)	þ	LncRNA H19 (mechanosensitive lncRNA enriched in exosomes, key mediator)	[14]
1. Histology (Safranin O/Fast Green, OARSI score) 2. IHC (Collagen II, Collagen I, VEGFA) 3. Micro-CT (BV/TV, Tb.Th, Tb.N, BMD) 4. Gross morphology scoring	þ	miR-140-5p (validated, targets VEGFA)	[15]
1. Histology (Safranin O/Fast Green staining) 2. Cartilage degeneration scoring (OARSI score) 3. Immunohistochemistry (Collagen II, MMP13, ADAMTS5) 4. TUNEL staining (apoptosis in cartilage)	þ	miR-26a-5p (targets PTEN/AKT pathway)	[16]
1. Histology (Safranin O/Fast Green, OARSI score) 2. IHC (Collagen II, Aggrecan, SOX9, MMP13, ADAMTS5) 3. Gait analysis	þ	miR-148a (exosomal enrichment, regulates Wnt/β-catenin)	[17]
1. Histology (Safranin O/Fast Green, OARSI score) 2. Micro-CT (osteophyte formation) 3. IHC/IF (Collagen II, Aggrecan, ADAMTS5, MMP13) 4. ELISA (IL-1β, IL-18, TNF-α)	þ	miR-1208 (targets METTL3 → reduces m6A of NLRP3)	[18]
1. Histology (Safranin O/Fast Green staining) 2. Cartilage degeneration scoring (OARSI score) 3. Immunohistochemistry (Collagen II, MMP13, ADAMTS5) 4. Micro-CT (subchondral bone changes)	þ	miR-136-5p (targets ELF3, regulates ECM metabolism)	[19]
1. Histology (Safranin O-Fast Green; OARSI score) 2. IHC/IF (COL2A1/COL II, MMP-13)3. TEM of autophagic vesicles; in vivo delivery images.	þ	miR-199a-3p (enriched in exosomes; functional cargo validated with delivery/antagomir and mTOR–autophagy readouts)	[20]
1. Function (weight-bearing test) 2. Histology (Safranin O-Fast Green with GAG quantification)3. IHC (ADAMTS5)	þ	Reported 7 candidate miRNAs in hypoxia-Exos by NGS; no single miRNA validated in vivo.	[21]
1. Histology (H&E; Toluidine Blue; Safranin O-Fast Green; Mankin score)2. IHC (COL II, COL I); IF (MMP-13, p53)3. Imaging (X-ray joint space, micro-CT) 4. Joint gross score/width.	x	NR	[22]
1. Imaging (micro-CT subchondral bone/osteophytes)2. Histology (cartilage & synovium changes)3. (paper focuses on TRPV4/osteoclast axis)	x	NR	[23]

Outcome index	EV cargo analysis	Specific proteins & miRNAs	Ref
1. Pain/Function (weight-bearing); Histopathology 2. IHC (pro-inflammatory/catabolic/apoptotic proteins, incl. COL-II, MMP-8/13, cleaved caspase-3 as per abbreviations/methods) 3. dose-response.	þ	Protein cargo composition	[24]
1. Histology (H&E, Safranin O-Fast Green, Alcian blue) 2. IHC (COL I, COL II) 3. Synovial fluid ELISA (IL-1β, IL-6, TNF-α); micro-CT (BV/TV, Ct.Th, Tb.N, Tb.Th, Tb.Sp, osteophyte volume)	x	NR	[25]
1. Histology (HE and Safranin O-FG; OARSI score, Mankin score) 2. IHC (Collagen II, MMP-13, ADAMTS-5) 3. miRNA Expression (let-7b targeting ADAMTS-5) 4. Gene Expression (COL2A1, ACAN, MMPs, inflammatory genes)	þ	miR-320a, miR-107, miR-320c, miR-137, miR-222, miR-27a, miR-125a-5p; proteins CD81, APOB, ITGAV, ITGB3, C3, SERPINE1, A2M; validated functional targets STAT1 and PPARγ	[26]
1. Histology (Safranin O, Alcian Blue staining; GAG/proteoglycan content)2. qPCR (COL2A1, COL1A1, COL10A1, ACAN, SOX9, MMP13, ADAMTS5, TNF-α, IL-6) 3. Weight-Bearing Analysis (functional assessment in OA rats) 4. miRNA Profiling (Exosomal miRNAs: miR-21, miR-29a, miR-92a, etc.)	þ	miRNA Profiling (Exosomal miRNAs: miR-21, miR-29a, miR-92a)	[27]
Histology (Safranin O, OARSI score); IHC (COL II, MMP-13); ELISA (IL-1β, TNF-α, IL-6 in serum/synovial fluid); Functional (paw withdrawal threshold, weight distribution)	x	NR	[28]
Histology (Safranin O, H&E, OARSI score); IHC (COL II, MMP-13, ADAMTS5); TUNEL (apoptosis); Micro-CT	þ	miR-140-5p functionally validated as cartilage-protective	[29]
Osteophyte Histology: Safranin O/fast green; OARSI score IHC: COL2A1, MMP13			[30]
Histology (Safranin O, OARSI scoring); IHC (COL II, MMP-13); Micro-CT (osteophyte volume, bone structure); Functional (pain threshold, gait)	þ	miR-26a-5p enriched and validated; PTEN/AKT axis	[31]
Histology (Safranin O, Mankin/OARSI scoring); IHC (COL II, MMP-13, Aggrecan); Micro-CT (subchondral bone parameters); Functional (gait analysis)	þ	miR-92a-3p identified and validated as protective cargo	[32]
Histology (Safranin O-Fast Green, OARSI score); IHC (COL II, MMP-13, Aggrecan); ELISA (inflammatory cytokines)	þ	miR-135b identified and validated (via Sp1/NF-κB axis)	[33]
Histology (Safranin O-Fast Green, H&E); IHC (COL II, MMP-13, ADAMTS5); TUNEL (apoptosis); Micro-CT (subchondral bone, osteophytes)	þ	miR-100-5p enriched; validated for mTOR/autophagy regulation	[34]
Histology (HE); TUNEL (apoptosis index); IHC (COL II, COL X, Runx2, Sox9); Flow cytometry (M1/M2 macrophage polarization)	þ	lncRNA TUC339 validated as functional exosomal cargo	[35]
Histology (Safranin O/Fast Green, FJ OA score); IHC (COL II, MMP-13, Aggrecan, OCN); SR-FTIR (collagen/proteoglycan distribution); 3D X-ray micro-CT (bone remodeling, H-type vessel formation); Pain (Von Frey, vocalization threshold)	x	Not assessed (focus on hypoxia conditioning of ADSC-Exos)	[36]
Histology (Safranin O-Fast Green); IHC (COL II, COL I, SOX9, RUNX2, MMP-13); Micro-CT (bone volume, trabecular structure); Pain assessment (weight-bearing, gait)	þ	circRNA cargos highlighted; specifically circRNA_0001236–miR-34a-5p axis validated	[37]

References

[1] Y. Zhu, Y. Wang, B. Zhao, X. Niu, B. Hu, Q. Li, J. Zhang, J. Ding, Y. Chen, Y. Wang. Comparison of exosomes secreted by induced pluripotent stem cell-derived mesenchymal stem cells and synovial membrane-derived mesenchymal stem cells for the treatment of osteoarthritis. *Stem Cell Research & Therapy* **8** (2017) 64. https://doi.org/10.1186/s13287-017-0510-9

[2] S.C. Tao, T. Yuan, Y.L. Zhang, W.J. Yin, S.C. Guo, C.Q. Zhang. Exosomes derived from miR-140-5p-overexpressing human synovial mesenchymal stem cells enhance cartilage tissue regeneration and prevent osteoarthritis of the knee in a rat model. *Theranostics* **7** (2017) 180-195. https://doi.org/10.7150/thno.17133

[3] Y. Wang, D. Yu, Z. Liu, F. Zhou, J. Dai, B. Wu, J. Zhou, B.C. Heng, X.H. Zou, H. Ouyang, H. Liu. Exosomes from embryonic mesenchymal stem cells alleviate osteoarthritis through balancing synthesis and degradation of cartilage extracellular matrix. *Stem Cell Research & Therapy* **8** (2017) 189. https://doi.org/10.1186/s13287-017-0632-0

[4] J. Wu, L. Kuang, C. Chen, J. Yang, W.N. Zeng, T. Li, H. Chen, S. Huang, Z. Fu, J. Li, R. Liu, Z. Ni, L. Chen, L. Yang. miR-100-5p-abundant exosomes derived from infrapatellar fat pad MSCs protect articular cartilage and ameliorate gait abnormalities via inhibition of mTOR in osteoarthritis. *Biomaterials* **206** (2019) 87-100. https://doi.org/10.1016/j.biomaterials.2019.03.022

[5] C.H. Woo, H.K. Kim, G.Y. Jung, Y.J. Jung, K.S. Lee, Y.E. Yun, J. Han, J. Lee, W.S. Kim, J.S. Choi, S. Yang, J.H. Park, D.G. Jo, Y.W. Cho. Small extracellular vesicles from human adipose-derived stem cells attenuate cartilage degeneration. *Journal of Extracellular Vesicles* **9** (2020) 1735249. https://doi.org/10.1080/20013078.2020.1735249

[6] X. Zhou, H. Liang, X. Hu, J.N. An, S. Ding, S. Yu, C. Liu, F. Li, Y. Xu. BMSC-derived exosomes from congenital polydactyly tissue alleviate osteoarthritis by promoting chondrocyte proliferation. *Cell Death Discovery* **6** (2020) 96. https://doi.org/10.1038/s41420-020-00374-z

[7] X. Xu, Y. Liang, X. Li, K. Ouyang, M. Wang, T. Cao, W. Li, J. Liu, J. Xiong, B. Li, J. Xia, D. Wang, L. Duan. Exosome-mediated delivery of kartogenin for chondrogenesis of synovial fluid-derived mesenchymal stem cells and cartilage regeneration. *Biomaterials* **269** (2021) 120539. https://doi.org/10.1016/J.BIOMATERIALS.2020.120539

[8] K. Wang, F. Li, Y. Yuan, L. Shan, Y. Cui, J. Qu, F. Lian. Synovial mesenchymal stem cell-derived EV-packaged miR-31 downregulates histone demethylase KDM2A to prevent knee osteoarthritis. *Molecular Therapy: Nucleic Acids* **22** (2020) 1078-1091. https://doi.org/10.1016/j.omtn.2020.09.014

[9] S. Tang, P. Chen, H. Zhang, H. Weng, Z. Fang, C. Chen, G. Peng, H. Gao, K. Hu, J. Chen, L. Chen, X. Chen. Comparison of curative effect of human umbilical cord-derived mesenchymal stem cells and their small extracellular vesicles in treating osteoarthritis. *International Journal of Nanomedicine* **16** (2021) 8185-8202. https://doi.org/10.2147/IJN.S336062

[10] H. Fazaeli, N. Kalhor, L. Naserpour, F. Davoodi, M. Sheykhhasan, S.K.E. Hosseini, M. Rabiei, A. Sheikholeslami. A comparative study on the effect of exosomes secreted by mesenchymal stem cells derived from adipose and bone marrow tissues in the treatment of osteoarthritis-induced mouse model. *BioMed Research International* **2021** (2021) 9688138. https://doi.org/10.1155/2021/9688138

[11] Y. Jin, M. Xu, H. Zhu, C. Dong, J. Ji, Y. Liu, A. Deng, Z. Gu. Therapeutic effects of bone marrow mesenchymal stem cells-derived exosomes on osteoarthritis. *Journal of Cellular and Molecular Medicine* **25** (2021) 9281-9294. https://doi.org/10.1111/JCMM.16860

[12] K.S. Lee, J. Lee, H.K. Kim, S.H. Yeom, C.H. Woo, Y.J. Jung, Y.E. Yun, S.Y. Park, J. Han, E. Kim, J.H. Sul, J.M. Jung, J.H. Park, J.S. Choi, Y.W. Cho, D.-G. Jo. Extracellular vesicles from adipose tissue-derived stem cells alleviate osteoporosis through osteoprotegerin and miR-21-5p. *Journal of Extracellular Vesicles* **10** (2021) e12152. https://doi.org/10.1002/jev2.12152

[13] A. Duan, K. Shen, B. Li, C. Li, H. Zhou, R. Kong, Y. Shao, J. Qin, T. Yuan, J. Ji, W. Guo, X. Wang, T. Xue, L. Li, X. Huang, Y. Sun, Z. Cai, W. Liu, F. Liu. Extracellular vesicles derived from LPS-preconditioned human synovial mesenchymal stem cells inhibit extracellular matrix degradation and prevent osteoarthritis of the knee in a mouse model. *Stem Cell Research & Therapy* **12** (2021) 427. https://doi.org/10.1186/s13287-021-02507-2

[14] L. Yan, G. Liu, X. Wu. Exosomes derived from umbilical cord mesenchymal stem cells in mechanical environment show improved osteochondral activity via upregulation of LncRNA H19. *Journal of Orthopaedic Translation* **26** (2021) 111-120. https://doi.org/10.1016/j.jot.2020.03.005

[15] Y. Liu, Y. Zeng, H.B. Si, L. Tang, H.Q. Xie, B. Shen. Exosomes derived from human urine-derived stem cells overexpressing miR-140-5p alleviate knee osteoarthritis through downregulation of VEGFA in a rat model. *American Journal of Sports Medicine* **50** (2022) 1088-1105. https://doi.org/10.1177/03635465221073991

[16] K. Li, G. Yan, H. Huang, M. Zheng, K. Ma, X. Cui, D. Lu, L. Zheng, B. Zhu, J. Cheng, J. Zhao. Anti-inflammatory and immunomodulatory effects of the extracellular vesicles derived from human umbilical cord mesenchymal stem cells on osteoarthritis via M2 macrophages. *Journal of Nanobiotechnology* **20** (2022) 38. https://doi.org/10.1186/S12951-021-01236-1

[17] Y.H. Hsueh, W. Buddhakosai, P.N. Le, Y.Y. Tu, H.C. Huang, H.E. Lu, W.L. Chen, Y.K. Tu. Therapeutic effect of induced pluripotent stem cell-derived extracellular vesicles in an in vitro and in vivo osteoarthritis model. *Journal of Orthopaedic Translation* **38** (2023) 141-155. https://doi.org/10.1016/j.jot.2022.10.004

[18] H. Zhou, X. Shen, C. Yan, W. Xiong, Z. Ma, Z. Tan, J. Wang, Y. Li, J. Liu, A. Duan, F. Liu. Extracellular vesicles derived from human umbilical cord mesenchymal stem cells alleviate osteoarthritis of the knee in mice model by interacting with METTL3 to reduce m6A of NLRP3 in macrophage. *Stem Cell Research & Therapy* **13** (2022) 322. https://doi.org/10.1186/S13287-022-03005-9

[19] P. Chen, S. Tang, H. Gao, H. Zhang, C. Chen, Z. Fang, G. Peng, H. Weng, A. Chen, C. Zhang, Z. Qiu, S. Li, J. Chen, L. Chen, X. Chen. Wharton's jelly mesenchymal stem cell-derived small extracellular vesicles as natural nanoparticles to attenuate cartilage injury via microRNA regulation. *International Journal of Pharmaceutics* **623** (2022) 121952. https://doi.org/10.1016/j.ijpharm.2022.121952

[20] S. Zhao, G. Xiu, J. Wang, Y. Wen, J. Lu, B. Wu, G. Wang, D. Yang, B. Ling, D. Du, J. Xu. Engineering exosomes derived from subcutaneous fat MSCs specially promote cartilage repair as miR-199a-3p delivery vehicles in osteoarthritis. *Journal of Nanobiotechnology* **21** (2023) 220. https://doi.org/10.1186/s12951-023-02086-9

[21] L.H. Chang, S.C. Wu, C.H. Chen, J.W. Chen, W.C. Huang, C.W. Wu, Y.S. Lin, Y.J. Chen, J.K. Chang, M.L. Ho. Exosomes derived from hypoxia-cultured human adipose stem cells alleviate articular chondrocyte inflammaging and post-traumatic osteoarthritis progression. *International Journal of Molecular Sciences* **24** (2023) 13414. https://doi.org/10.3390/ijms241713414

[22] H. Cao, M. Chen, X. Cui, Y. Liu, Y. Liu, S. Deng, T. Yuan, Y. Fan, Q. Wang, X. Zhang. Cell-free osteoarthritis treatment with sustained-release of chondrocyte-targeting exosomes from umbilical cord-derived mesenchymal stem cells to rejuvenate aging chondrocytes. *ACS Nano* **17** (2023) 13358-13376. https://doi.org/10.1021/acsnano.3c01612

[23] Y. Fu, S. Cui, Y. Zhou, L. Qiu. Dental pulp stem cell-derived exosomes alleviate mice knee osteoarthritis by inhibiting TRPV4-mediated osteoclast activation. *International Journal of Molecular Sciences* **24** (2023) 4926. https://doi.org/10.3390/ijms24054926

[24] C. Huang, Y. Zhao, S. Lin, L. Li, X. Guo, S. Yumiseba, J. Yang, R. Hariri, Q. Ye, S. He, A. Kilcoyne. Characterization of human placenta-derived exosome (pExo) as a potential osteoarthritis disease modifying therapeutic. *Arthritis Research & Therapy* **25** (2023) 218. https://doi.org/10.1186/s13075-023-03219-z

[25] X. Pan, X. Li, L. Zhang, F. Wu, Q. Zhang, S. Xu, C. Shen, J. Liang, R. Pan. Umbilical cord mesenchymal stem cells relieve osteoarthritis in rats through immunoregulation and inhibition of chondrocyte apoptosis. *Scientific Reports* **13** (2023) 15779. https://doi.org/10.1038/s41598-023-42349-x

[26] H. Yang, Y. Zhou, B. Ying, X. Dong, Q. Qian, S. Gao. Effects of human umbilical cord mesenchymal stem cell-derived exosomes in the rat osteoarthritis models. *Stem Cells Translational Medicine* **13** (2024) 803-811. https://doi.org/10.1093/stcltm/szae031

CC BY

[27] A.I. Figueroa-Valdés, P. Luz-Crawford, Y. Herrera-Luna, N. Georges-Calderón, C. García, H.E. Tobar, M.J. Araya, J. Matas, D. Donoso-Meneses, C. de la Fuente, J. Cuenca, E. Parra, F. Lillo, C. Varela, M.I. Cádiz, R. Vernal, A. Ortloff, G. Nardocci, V. Castañeda, … F. Alcayaga-Miranda. Clinical-grade extracellular vesicles derived from umbilical cord mesenchymal stromal cells: preclinical development and first-in-human intra-articular validation as therapeutics for knee osteoarthritis. *Journal of Nanobiotechnology* **23** (2025) 13. https://doi.org/10.1186/s12951-024-03088-x

[28] P. Li, S. Lv, W. Jiang, L. Si, B. Liao, G. Zhao, Z. Xu, L. Wang, J. Zhang, H. Wu, Q. Peng, Z. Li, L. Qi, G. Chi, Y. Li. Exosomes derived from umbilical cord mesenchymal stem cells protect cartilage and regulate the polarization of macrophages in osteoarthritis. *Annals of Translational Medicine* **10** (2022) 976. https://doi.org/10.21037/ATM-22-3912

[29] S. Cosenza, M. Ruiz, K. Toupet, C. Jorgensen, D. Noël. Mesenchymal stem cells derived exosomes and microparticles protect cartilage and bone from degradation in osteoarthritis. *Scientific Reports* **7** (2017) 16214. https://doi.org/10.1038/s41598-017-15376-8

[30] J. Li, Z. Ding, Y. Li, W. Wang, J. Wang, H. Yu, A. Liu, J. Miao, S. Chen, T. Wu, Y. Cao. BMSCs-derived exosomes ameliorate pain via abrogation of aberrant nerve invasion in subchondral bone in lumbar facet joint osteoarthritis. *Journal of Orthopaedic Research* **38** (2020) 670-679. https://doi.org/10.1002/jor.24497

[31] J. Zhang, Y. Rong, C. Luo, W. Cui. Bone marrow mesenchymal stem cell-derived exosomes prevent osteoarthritis by regulating synovial macrophage polarization. *Aging* **12** (2020) 25138-25152. https://doi.org/10.18632/aging.104110

[32] Y. Jin, Z. Ren, S. Qi. Exosomal miR-9-5p secreted by bone marrow-derived mesenchymal stem cells alleviates osteoarthritis by inhibiting syndecan-1. *Cell and Tissue Research* **381** (2020) 99-114. https://doi.org/10.1007/s00441-020-03193-x

[33] L. He, T. He, J. Xing, Q. Zhou, L. Fan, C. Liu, Y. Chen, D. Wu, Z. Tian, B. Liu, L. Rong. Bone marrow mesenchymal stem cell-derived exosomes protect cartilage damage and relieve knee osteoarthritis pain in a rat model of osteoarthritis. *Stem Cell Research & Therapy* **11** (2020) 276. https://doi.org/10.1186/s13287-020-01781-w

[34] H. Xu, B. Xu. BMSC-derived exosomes ameliorate osteoarthritis by inhibiting pyroptosis of cartilage via delivering miR-326 targeting HDAC3 and STAT1/NF-κB p65 to chondrocytes. *Mediators of Inflammation* **2021** (2021) 9972805. https://doi.org/10.1155/2021/9972805

[35] J. Hu, Z.S. Shi, X.Z. Liu, H.T. Cai, A.F. Yang, D.M. Sun, L.L. Xu, Y. Yang, Z.H. Li. Exosomes derived from Runx2-overexpressing BMSCs enhance cartilage tissue regeneration and prevent osteoarthritis of the knee in a rabbit model. *Stem Cells International* **2022** (2022) 6865041. https://doi.org/10.1155/2022/6865041

[36] J. Zhao, Y. Sun, X. Sheng, J. Xu, G. Dai, R. He, Y. Jin, Z. Liu, Y. Xie, T. Wu, Y. Cao, J. Hu, C. Duan. Hypoxia-treated adipose mesenchymal stem cell-derived exosomes attenuate lumbar facet joint osteoarthritis. *Molecular Medicine* **29** (2023) 118. https://doi.org/10.1186/s10020-023-00709-3

[37] X. Shen, J. Qin, Z. Wei, F. Liu. Bone marrow mesenchymal stem cell exosome-derived lncRNA TUC339 influences the progression of osteoarthritis by regulating synovial macrophage polarization and chondrocyte apoptosis. *Biomedicine & Pharmacotherapy* **167** (2023) 115488. https://doi.org/10.1016/j.biopha.2023.115488.

[38] B. Li, E. Shen, Z. Wu, H. Qi, D. Liu, X. Jiang. BMSC-derived exosomes attenuate rat osteoarthritis by regulating macrophage polarization through PINK1/Parkin signaling pathway. *Cartilage* **15** (2024) 1-10. https://doi.org/10.1177/19476035241245805.
